# Activated charcoal filtration profoundly alters systemic noradrenaline concentrations during hepatic chemosaturation: A prospective cohort study

**DOI:** 10.1371/journal.pone.0350771

**Published:** 2026-08-31

**Authors:** Jan Turra, Dania Fischer, Osman Öcal, Oliver Biecker, Philipp Mayer, Philippe Grieshaber, Folker Wenzel, Matthias Karck, Marina Nikolic, Maik von der Forst, Markus Weigand, Hans-Ulrich Kauczor, Jessica Cecile Hassel, Christoph Lichtenstern, Maximilian Dietrich, Patrick Rehn

**Affiliations:** 1 Department of Perfusion, Heidelberg University Hospital, Im Neuenheimer Feld, Heidelberg, Germany; 2 Department of Anesthesiology, Heidelberg University Hospital, Im Neuenheimer Feld, Heidelberg, Germany; 3 Department of Radiology, Heidelberg University Hospital, Im Neuenheimer Feld, Heidelberg, Germany; 4 Department of Pediatric Cardiac Surgery, University Hospital Münster, Albert-Schweitzer-Campus 1, Münster, Germany; 5 Furtwangen University of Applied Sciences, Faculty II: Engineering and Technology, Robert-Gerwig-Platz 1, Furtwangen im Schwarzwald, Germany; 6 Department of Cardiac Surgery, University Hospital Heidelberg, Im Neuenheimer Feld 420, Heidelberg, Germany; 7 Heidelberg University, Medical Faculty Heidelberg, Department of Dermatology and National Center for Tumor Diseases (NCT), NCT Heidelberg, a partnership between DKFZ and University Hospital Heidelberg, Heidelberg, Germany; Sichuan University, CHINA

## Abstract

**Background:**

Hepatic chemosaturation using extracorporeal circulation (ECC) is associated with severe hemodynamic instability. Activated charcoal filters are used to prevent systemic toxicity, but their effects on circulating catecholamines remain poorly understood.

**Methods:**

In this prospective, single-center observational study, systemic catecholamine concentrations were measured in 15 procedures undergoing hepatic chemosaturation. Blood samples were obtained at predefined time points: after induction of anesthesia (T0), during initiation of ECC (T1), immediately before (T2.1) and after charcoal filtration (T2.2) and after termination of ECC (T3).

**Results:**

Systemic noradrenaline concentrations changed significantly over time (p < 0.001). The measured noradrenaline concentration increased markedly during inferior vena cava occlusion and ECC. This was consistent with pronounced hemodynamic instability and a significant increase in the dose of noradrenaline administered (T1 and T2.1 vs. T0), followed by a near-complete reduction across the activated charcoal filter (median extraction rate 95.5%) with continued high NA administration (median 0.35 µg/kg/min). In contrast, adrenaline and dopamine concentrations remained stable and were neither administered nor significantly affected by filtration. After termination of ECC, a pronounced rebound increase in noradrenaline concentrations was observed. These biochemical changes were accompanied by metabolic acidosis and rising lactate levels.

**Conclusion:**

Activated charcoal filtration during hepatic chemosaturation selectively removes circulating noradrenaline and is a key contributor to perioperative hemodynamic instability. Awareness of this mechanism is essential for tailored vasopressor strategies and safer anesthetic management.

**Trial registration:**

DRKS, DRKS00034527. Registered 24 June 2024, https://drks.de/search/de/trial/DRKS00034527

## Introduction

Hepatic chemosaturation using extracorporeal circulation (ECC) has emerged as an important therapeutic option for patients with unresectable primary or secondary liver malignancies. This technique enables the delivery of high-dose chemotherapy directly to the liver while substantially reducing systemic exposure and associated toxicity, thereby representing a valuable palliative treatment strategy [1,2]. The system comprises intravascular catheters and an ECC circuit equipped with an activated carbon filter for hemofiltration. The chemotherapeutic agent most used is melphalan hydrochloride, which has demonstrated high efficacy in the treatment of both primary hepatic malignancies and liver metastases from various primary tumors, while being associated with reversible hepatic toxicity [3–7]. The chemotherapeutic agent is administered selectively into the hepatic arterial branches supplying the tumor via a microcatheter inserted through femoral arterial access. Hepatic venous isolation is achieved using a double-balloon catheter introduced via femoral venous access. The cephalad balloon is positioned at the junction of the inferior vena cava (IVC) and the right atrium (RA), while the caudal balloon is placed infrahepatically within the IVC, thereby isolating hepatic venous outflow between the two balloons. Melphalan-saturated hepatic venous blood is aspirated through the double-balloon catheter and directed into the ECC. Within the ECC circuit, the blood passes through two activated carbon filters, which remove the chemotherapeutic agent. The purified blood is subsequently returned to the patient’s systemic circulation via the right internal jugular vein (IJV) [8]. Despite its oncological benefits, the procedure is regularly associated with pronounced perioperative hemodynamic instability and shock, posing a major challenge in perioperative hemodynamic management. Episodes of severe hypotension, high vasopressor requirements, and metabolic derangements are commonly observed, particularly during phases of ECC and active hemoperfusion using the activated charcoal filters. While these filters are effective in reducing systemic chemotherapeutic toxicity, they may also unintentionally adsorb endogenous and exogenous vasoactive substances [9–13]. Accordingly, the observed hemodynamic alterations are consistent with distributive shock physiology [10]. From an anesthesiologic perspective, this raises the clinically relevant concern that hemodynamic instability during chemosaturation may not solely reflect surgical stress or volume shifts but may in part be driven by filter-mediated removal of key regulatory mediators. Catecholamines play a central role in maintaining vascular tone and cardiac output (CO) during major surgical stress and ECC [14]. Noradrenaline represents the cornerstone of perioperative vasopressor therapy [15,16]. However, data on systemic catecholamine kinetics during hepatic chemosaturation and on the substance-specific effects of activated charcoal filtration remain scarce.

This study aimed to characterize systemic catecholamine dynamics during hepatic chemosaturation with ECC, with particular emphasis on the effects of activated charcoal filtration. We hypothesized that charcoal filters, intended to eliminate melphalan, also reduce circulating catecholamine levels and thereby cause the regularly observed intraoperative circulatory failure with shock. The results are intended to provide a pathophysiological basis for procedure-specific hemodynamic management.

## Methods

### Study design and population

We conducted a prospective, single-center exploratory study at University Hospital Heidelberg, Germany. Ethical approval for this study (reference number S214/2024) was provided by the Ethical Committee of the Medical Faculty of Heidelberg University (Chairperson Prof. Dr. med. Dr. h.c. Thomas Strowitzki) on 22 May 2024. Written informed consent was obtained for each participant prior to study inclusion. The study was registered with the German Clinical Trials Registry (DRKS ID: DRKS00034527). The study was conducted according to the Declaration of Helsinki. All patients scheduled for percutaneous chemosaturation of the liver at Heidelberg University Hospital, from from 1 September 2024 to 30 November 2025 were screened. Inclusion criteria were as follows: *(1) elective chemosaturation (2)* age ≥ 18 years*.* Exclusion criteria were: (1) emergency interventions (2) termination of the intervention due to a patent foramen ovale (3) pre-or intraoperative hemodynamic instability resulting in the termination of the procedure (4) pre-existing pheochromocytoma. According to local standards, patients with severe cardiac, pulmonary, hepatic, or renal comorbidities were not eligible to receive chemosaturation. In each procedure, serum levels of noradrenaline, adrenaline, and dopamine were measured at anesthesia induction (T0), ECC induction and balloon occlusion (T1), during ECC (T2) and after ECC termination (T3). Of note, at T2 two blood samples were taken simultaneously: one before and one after the activated charcoal filter. Catecholamines were measured in the central laboratory, using High-Performance Liquid Chromatography (HPLC). Additionally, blood gas analyses (RAPIDPoint 500 System®, Siemens) were done at all timepoints. All relevant patient data were collected prospectively. The recorded data included epidemiological data, indication for chemosaturation, comorbidities, laboratory values, catecholamine dose and amount of fluid given intra-interventionally. Values were taken at the mentioned timepoints. Catecholamine dose was recorded as a continuous flow rate. At the predefined timepoints of chemosaturation, the highest catecholamine flow rate was recorded in each procedure. For mean arterial pressure (MAP), we recorded the lowest value, and for HR, the highest value for each timepoint. No a priori sample size calculation was performed given the exploratory nature of the study. All eligible chemosaturation procedures performed during the study period were consecutively included.

### Procedure

**Fig 1:**
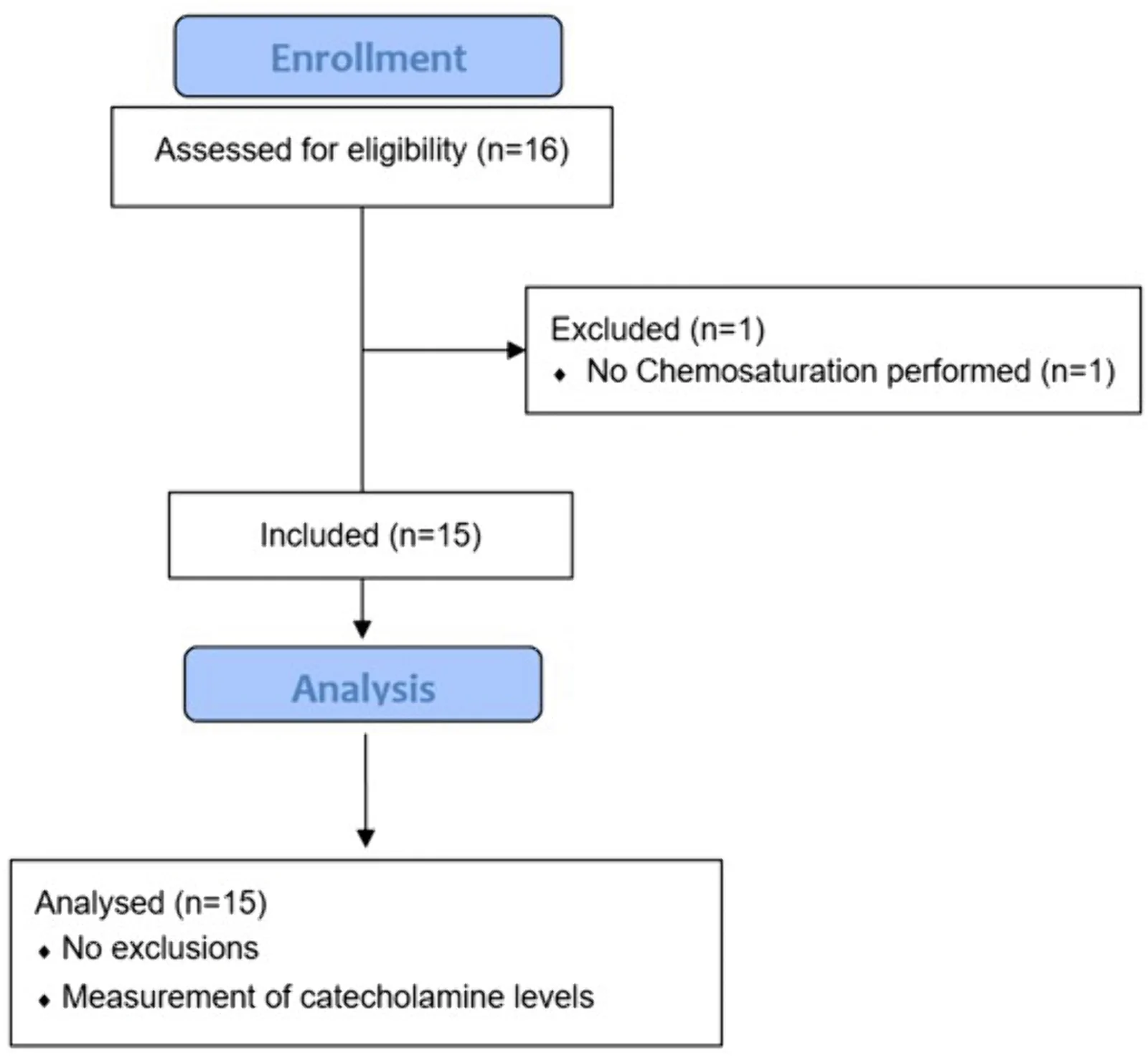
Consort diagram for enrollment of chemosaturation procedures.

First, patients were placed under general anesthesia using induction agents propofol (Fresenius Kabi GmbH® (Bad Homburg, Germany)), sufentanil (Hameln Pharma GmbH® (Hameln, Germany)), and rocuronium (B. Braun SE® (Melsungen, Germany)) and endotracheally intubated. General anesthesia was maintained using a balanced approach with sevoflurane (AbbVie GmbH & Co. KG® (Wiesbaden, Germany)) and a continuous infusion of remifentanil (Hameln Pharma GmbH®) in all patients. A central venous line and an arterial line were placed in every patient. Prior to the start of the procedure, every patient was given peri-interventional antibiotic prophylaxis using 1.5 g cefuroxime as a single shot. Patent foramen ovale was ruled out by transesophageal echocardiography (TEE). The chemosaturation procedure was done by two interventional radiologists using the CHEMOSAT®-Catheter and CHEMOSAT® activated charcoal filtration system (Delcath Systems, Inc., New York, NY, USA) using melphalan as the chemotherapeutic agent. The priming volume of the ECC system consisted of approximately 700 ml of crystalloid solution and 5000 IU of heparin. After cannulation of a femoral artery and the contralateral femoral vein, a bolus of 400 IU/kg heparin was administered to achieve an Activated Clotting Time (ACT) > 450 s. If the ACT was < 450 s, a second bolus of 100–200 IU/kg heparin was administered, and the ACT was checked again. Filtration was performed by using ECC operated by a perfusionist. Blood flow was set to 500 ml/min in line with current recommendations by the manufacturer [17]. During ECC ACT was checked every 30 minutes to account for possible heparin washout. If the ACT approached 400s or fell below 400s another heparin bolus of 100 IU/kg was administered and ACT was checked again. During balloon occlusion, venous return from the entire infra-occlusion compartment (lower extremities, abdomen, and splanchnic and renal beds) reached the right atrium exclusively via the extracorporeal circuit at this flow rate. A schematic representation of the chemosaturation system setup can be found in Fig 1. Hemodynamic stabilization during the procedure was done by using fluid boluses of balanced crystalloid solution, continuous administration of noradrenaline and vasopressin, and boluses of noradrenaline as needed. Neither adrenaline nor dopamine was given exogenously. The reversal of the heparin’s effect after ECC was done by peripheral infusion of protamine in a dose of 200 IU/kg with a fixed flow rate of 200 mL/h and a goal of ACT < 160 s.

### Primary outcome

The primary outcome was the within-procedure change in catecholamine concentrations across the activated charcoal filter, quantified by paired pre-filter and post-filter plasma levels of noradrenaline, adrenaline, and dopamine during ECC (T2.1 vs. T2.2).

### Secondary outcomes

Secondary outcomes included:

- Changes in systemic catecholamine concentrations across predefined procedural time points (T0–T3).
- Peri-procedural hemodynamics, assessed by changes in MAP, central venous pressure (CVP), heart rate (HR), pulse pressure variation (PPV) and perfusion index (PI)
- Vasopressor and fluid requirements, quantified as administered doses of noradrenaline and vasopressin and total administered crystalloid volume.
- Metabolic consequences of ECC, evaluated by arterial pH, base excess, and lactate concentrations.

### Statistical methods

Continuous variables are reported as median and interquartile range (IQR). Categorical variables are reported as absolute and relative frequencies. Differences in catecholamine levels before and after filtration were analyzed using non-parametric methods. For comparisons across multiple time points, the Friedman test was applied. For pairwise comparisons between pre- and post-filtration measurements, the Wilcoxon signed-rank test was used. To estimate the magnitude of the paired differences, the Hodges–Lehmann estimator with corresponding 95% confidence intervals (CI) was calculated. Analyses were performed using R (version 4.5.1) and SPSS (IBM®, Version 30). A p-value <0.05 was considered statistically significant.

This study was designed as an exploratory cohort study. The trans-filter change in catecholamine concentrations (T2.1 vs. T2.2) was defined as the primary outcome. All other analyses were considered secondary and hypothesis-generating. Accordingly, no formal adjustment for multiple comparisons was applied to the secondary outcomes.

### Usage of artificial intelligence tools

As authors utilizing Generative AI and AI-assisted technologies we recognize the ethical responsibilities associated with these tools. In accordance with best practices and principles of scientific integrity, we hereby declare that during the preparation of this work, the authors utilized ChatGPT 5.2 (OpenAI, San Francisco, USA) program to revise grammar and enhance language coherence. Following the utilization of these tools, the authors meticulously reviewed and edited the content to ensure its compliance with the requirements. Consequently, the authors assume full responsibility for the integrity and accuracy of the publication.

## Results

### Patient characteristics

**Fig 2:**
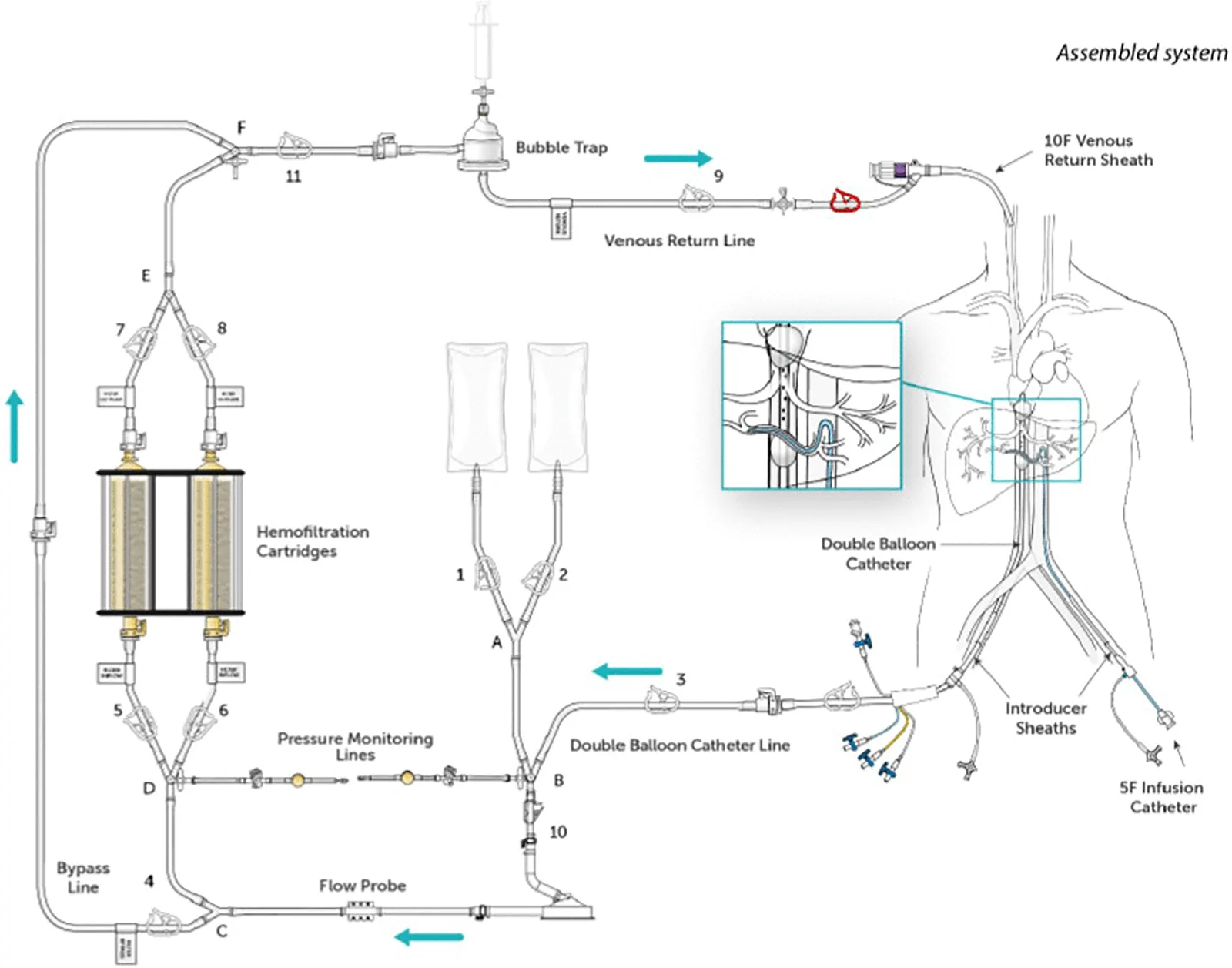
Schematic representation of chemosaturation setup. Image courtesy by Delcath Systems Inc.

We included 15 chemosaturation procedures in 12 individual patients in the analysis. All procedures included were performed in patients with uveal melanoma. This reflects the indication spectrum referred for chemosaturation at our center during the study period rather than a formal inclusion criterion. One patient was excluded after initial recruitment prior to the start of data collection because the procedure was not performed due to brain metastases (Fig 2). Baseline patient characteristics are summarized in Table 1. Median age was 62 years (IQR 43–66), and 53.3% of procedures were performed in male patients. Most patients were classified as American Society of Anesthesiologists Physical Status Classification (ASA) III (86.7%). Arterial hypertension was the most prevalent comorbidity (40%), while no patient had documented coronary artery disease (CAD) or chronic kidney disease (CKD).

**Table 1:** Baseline characteristics, BMI: Body Mass Index, ASA-Score: American Society of Anesthesiologists Physical Status Classification System, CAD: Coronary Artery Disease, CKD: Chronic Kidney Disease.

Patient-characteristics	Overall (n = 15 procedures)
**Age [year], median (IQR)**	**62 (43–66)**
**Sex [male], n (%)**	**8 (53.3)**
**BMI [kg/m²], median (IQR)**	**23.4 (21.6–25.8)**
**Uveal Melanoma n (%)**	**15 (100)**
**ASA-Score, n (%)** • **I** • **II** • **III** • **IV**	**0 (0)** **1 (6.7)** **13 (86.7)** **1 (6.7)**
**Comorbidities n (%)** • **Arterial Hypertension** • **CAD** • **CKD** • **Diabetes** **cerebrovascular disease**	**6 (40)** **0 (0)** **0 (0)** **2 (13.3)** **1 (6.7)**

### Systemic catecholamine dynamics during chemosaturation

**Fig 3:**
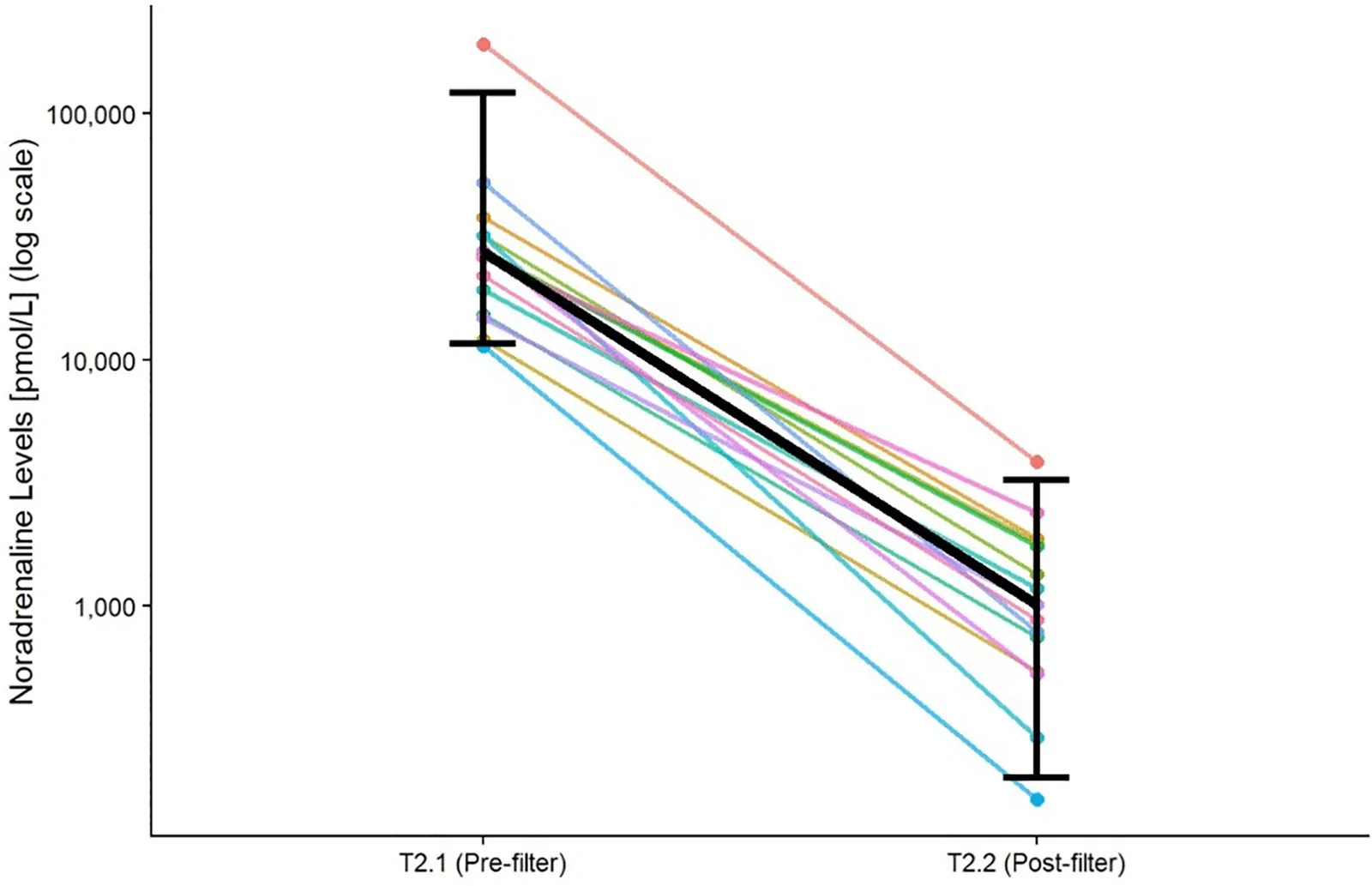
Levels of noradrenaline pre (T2.1) and post filter (T2.2). Each colored line represents one individual procedure. The bold black line indicates the overall median with interquartile range.

**Fig 4:**
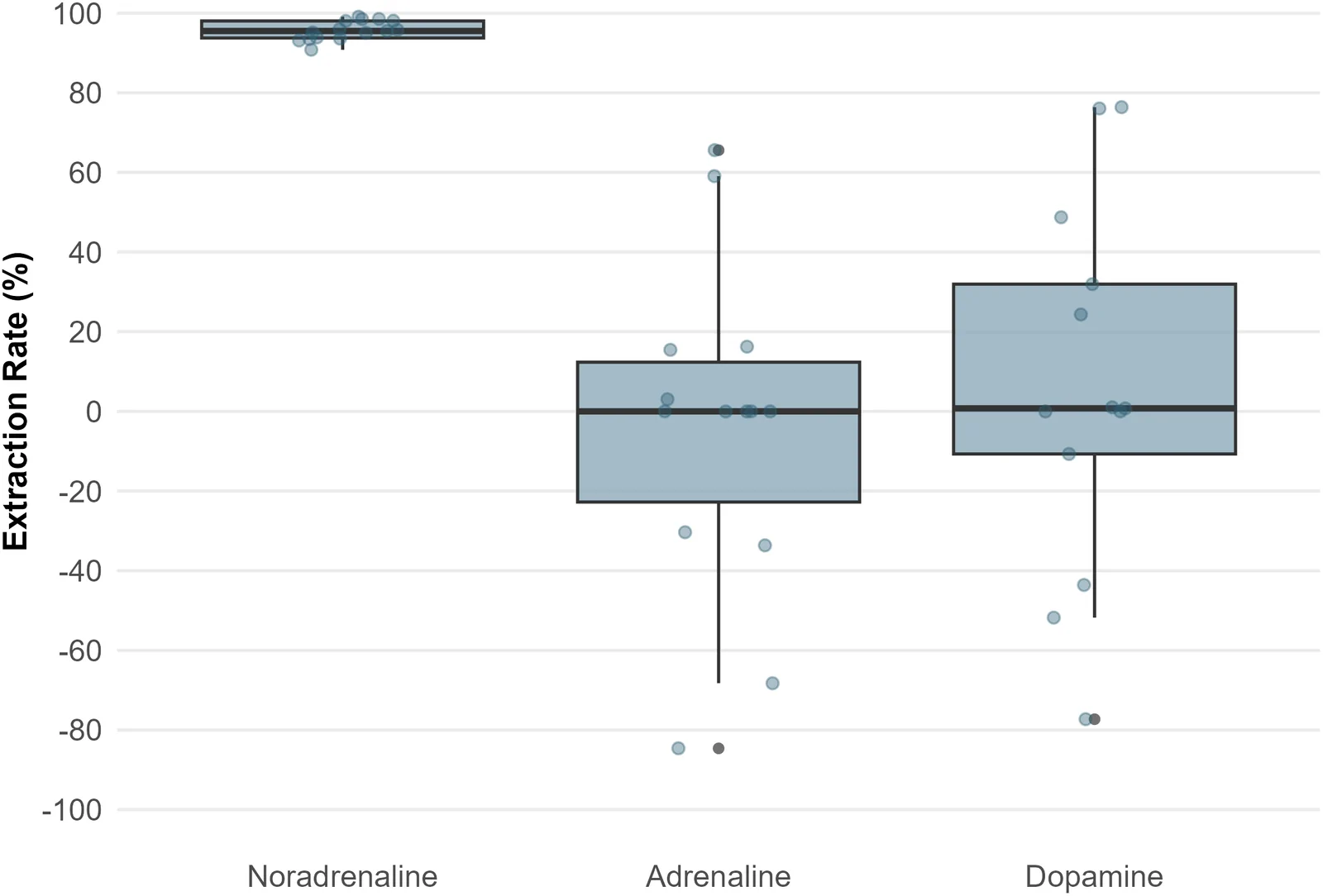
Extraction rate of Noradrenaline, Adrenaline and Dopamine across activated charcoal filter (T2.1 and T2.2).

Systemic noradrenaline concentrations changed significantly (p < 0.001, Table 2). In contrast, systemic adrenaline and dopamine concentrations remained stable throughout the procedure (Table 2). Neither adrenaline nor dopamine showed statistically significant changes across time points (p = 0.355 and p = 0.684, respectively). Direct paired pre- and post-filter measurements at T2.1 and T2.2 demonstrated a profound and selective adsorption effect of the activated carbon filter on noradrenaline (Table 3). Despite substitution for hemodynamic stability, median noradrenaline concentration decreased from 27404 pmol/L (IQR 15294 - 32000 pmol/L) pre-filter to 1017 pmol/L (IQR 544 - 1799 pmol/L) post-filter, corresponding to a Hodges–Lehmann median difference of −24901 pmol/l (95% CI -34737 to -18677; p < 0.001). There was a near complete elimination of systemic noradrenaline post filter (median extraction rate of 95.5% with IQR 93.6% − 98.1%, Figs 3 and 4). The trans-filter noradrenaline extraction was consistent across procedures, with a reduction observed in all 15 procedures and a minimum extraction of 90.8%. No significant differences were observed for adrenaline or dopamine across the filter (median fractional reduction for adrenaline and dopamine of 0%, Fig 4). Median concentrations of both catecholamines remained unchanged, and Wilcoxon signed-rank testing confirmed the absence of a relevant adsorption effect (adrenaline p = 0.646; dopamine p = 0.972).

**Table 2:** Systemic catecholamine concentrations during chemosaturation procedures, T0: anesthesia induction, T1: induction of ECC, T2.1: during ECC pre filter, T2.2: during ECC post filter, T3: after termination of ECC.

	T0	T1	T2.1 prefilter	T2.2 postfilter	T3	p-value (Friedman)
**Noradrenaline [pmol/l], median (IQR)**	2253 (1203–7454)	10439 (7332–17577)	27404 (15294–32000)	1017 (544–1799)	41297 (28986–126387)	<0.001
**Adrenaline [pmol/l], median (IQR)**	451 (273–644)	273 (273–518)	274 (273–504)	273 (273–504)	273 (273–711)	0.355
**Dopamine [pmol/l], median (IQR)**	611 (392–1073)	392 (392–851)	448 (392–999)	392 (392–695)	710 (392–918)	0.684

**Table 3:** Changes in systemic catecholamine concentrations pre- and postfilter (T2.1 and T2.2). HL: Hodges-Lehmann median difference, IQR: Interquartile range.

	Median (IQR) T2.1	Median (IQR) T2.2	HL median difference	95% CI	p-value (Wilcoxon)
**Noradrenaline [pmol/l]**	27404 (15294–32000)	1017 (544–1799)	−24901	−34737 – −18677	<0.001
**Adrenaline [pmol/l]**	274 (273–504)	273 (273–504)	50	−243–256	0.646
**Dopamine [pmol/l]**	448 (392–999)	392 (392–695)	3	−412–443	0.972

### Hemodynamic changes during chemosaturation

**Fig 5:**
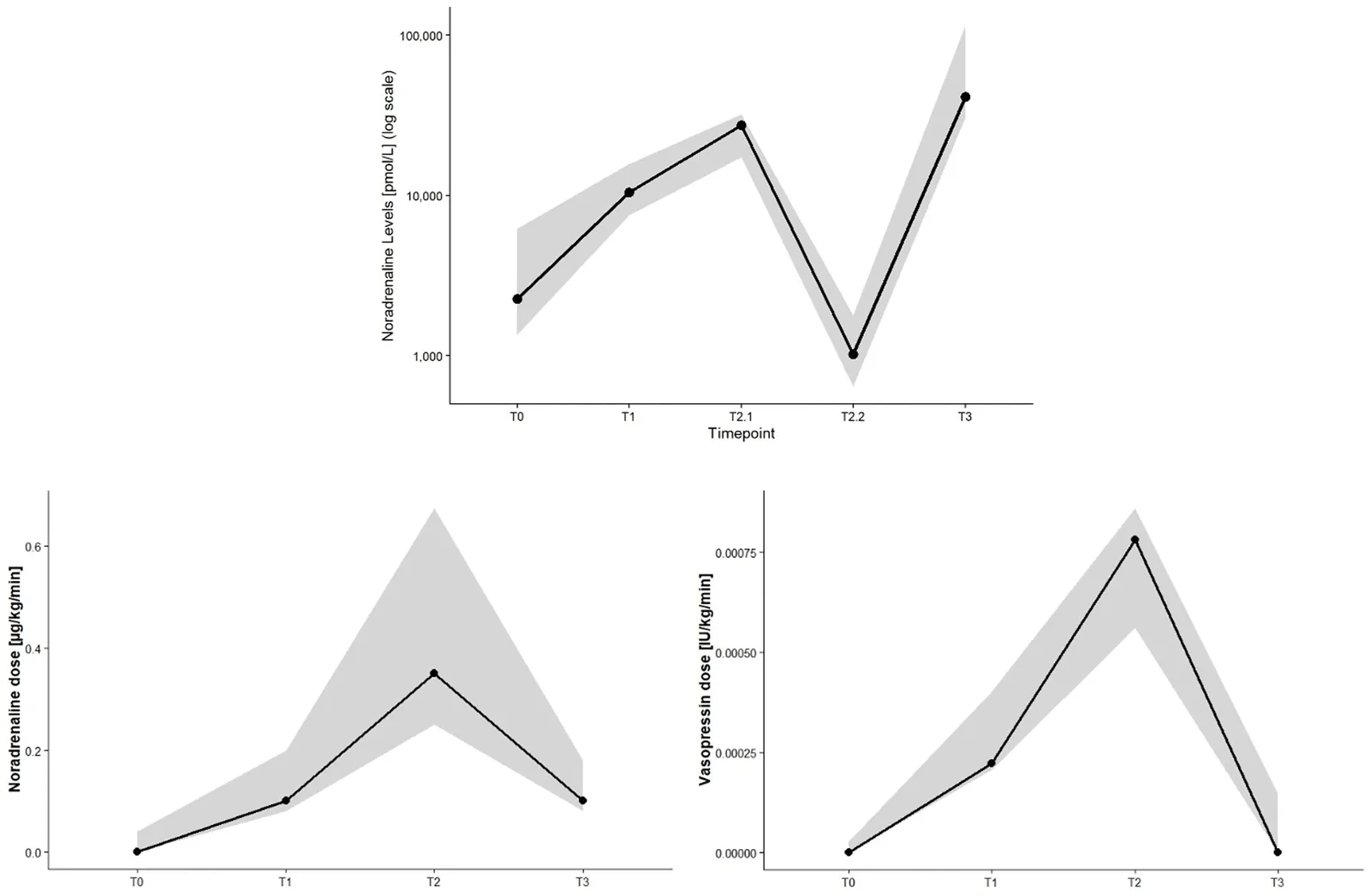
Noradrenaline levels across timepoints and corresponding externally administered doses of noradrenaline and vasopressin. Bold line indicated median, shaded area indicates interquartile range. T0: anesthesia induction, T1: induction of extracorporeal circulation (ECC), T2.1: during ECC pre filter, T2.2: during ECC post filter, T3: after termination of ECC.

Hemodynamic variables demonstrated significant alterations over time, particularly during ECC and filter activation (Table 4). MAP increased from T0 to T1, followed by a decrease during ECC with filter activation (T2), before partially recovering at T3 (p < 0.001). HR increased substantially at T2, reaching a median of 101 bpm, and subsequently decreased after ECC termination (p < 0.001). CVP reached a maximum at T3 (median 16 mmHg). Perfusion Index (PI) substantially decreased at T2 (median 0.7%). PPV showed a marked increase during T2 (median 24%), with normalization after ECC termination (p < 0.001). The observed hemodynamic instability was paralleled by a significant escalation in vasopressor therapy. Noradrenaline infusion rates increased from a median of 0.1 µg/kg/min at T1 to 0.35 µg/kg/min during T2, before decreasing again at T3 (p < 0.001). Vasopressin requirements followed a similar pattern, peaking during active filtration T2 (p = 0.002, Table 4 and Fig 5). Crystalloid administration increased progressively throughout the procedure, with the highest volumes given at T3 (median 1600 ml; p = 0.003). Concurrently, metabolic derangements developed during ECC, including significant acidosis and rising lactate levels. Median pH decreased to 7.28 at T2 and remained 7.29 at T3, while lactate concentrations increased, reaching a median of 26.4 mg/dl at T3 (all p < 0.001).

**Table 4:** Hemodynamic variables throughout the chemosaturation procedures. MAP: Mean arterial pressure, HR: heart rate, PPV: Pulse pressure variation, CVP: central venous pressure, PI: perfusion index, BE: Base excess, IQR: Interquartile range. T0: anesthesia induction, T1: induction of extracorporeal circulation (ECC), T2: during ECC, T3: after termination of ECC.

	T0	T1	T2	T3	p-value (Friedman)
**MAP [mmHg], median (IQR)**	71 (67–73)	106 (90–113)	80 (73–95)	72 (68–88)	<0.001
**HR [bpm], median (IQR)**	65 (56–69)	60 (55–64)	101 (81–118)	79 (70–91)	<0.001
**PPV [%],median (IQR)**	8 (5–9)	5 (2–5)	24 (19–40)	6 (4–8)	<0.001
**CVP [mmHg], median (IQR)**	12 (8–16)	15 (12–17)	12 (10–15)	16 (12–19)	0.002
**PI [%], median (IQR)**	2.6 (2–4.7)	2.1 (1.7–3.4)	0.7 (0.4–1.3)	3.1 (1.7 −4.0)	<0.001
**Noradrenaline support [µg/kg/min], median (IQR)**	0 (0–0.04)	0.1 (0.08–0.2)	0.35 (0.2–0.8)	0.1 (0.07–0.2)	<0.001
**Vasopressin [IE/kg/min], median (IQR)**	0 (0–0.00006)	0.0002 (0.0002–0.0005)	0.0008 (0.0005–0.0009)	0.0002 (0.0002–0.0003)	0.002
**Crystalloids [ml], median (IQR)**	500 (300–800)	300 (200–600)	1000 (500–1900)	1600 (1000–2300)	0.003
**pH, median (IQR)**	7.39 (7.36–7.42)	7.38 (7.33–7.40)	7.28 (7.23–7.29)	7.29 (7.21–7.32)	<0.001
**BE, median (IQR)**	−2.7 (−4.4 – −0.6)	−3.8 (−5.7 – −2.0)	−8.1 (−10.4 – −6.6)	−6.9 (−9.4 – −4.0)	<0.001
**Lactate [mg/dl], median (IQR)**	9.4 (6.4–12.3)	9.7 (8.2–13.4)	12.2 (9.1–18.4)	26.4 (18.8–39.4)	<0.001

## Discussion

Hepatic chemosaturation is frequently complicated by pronounced peri-procedural hemodynamic instability [18]. This study is the first to systematically investigate its underlying mechanisms in a larger human cohort. We demonstrate that the activated charcoal filter selectively and almost completely removes circulating noradrenaline during ECC, providing a plausible pathophysiological explanation for the observed hemodynamic derangements.

This mechanistic finding is of particular relevance given the central role of noradrenaline in maintaining vascular tone and systemic vascular resistance during major procedural stress and ECC [14–16]. Our data show that both endogenously released and exogenously administered noradrenaline are subject to near-complete adsorption by the activated charcoal filter, with extraction rates exceeding 95%. This profound depletion coincides temporally with the phase of maximal hemodynamic instability, characterized by hypotension, tachycardia, increased pulse pressure variation, and escalating vasopressor requirements [10]. Importantly, despite increasing infusion rates, post-filter systemic noradrenaline concentrations remained low, suggesting immediate and ongoing removal within the extracorporeal circuit and providing a mechanistic explanation for the frequently observed catecholamine “resistance” during active filtration [8,10,12,19].

A key observation of this study is the apparent selectivity of catecholamine adsorption. In contrast to noradrenaline, circulating adrenaline and dopamine concentrations remained stable throughout the procedure and showed no relevant trans-filter gradient. Two complementary explanations should be considered. First, this apparent selectivity is to a relevant extent a consequence of the markedly different baseline concentrations of the three catecholamines. Pre-filter noradrenaline concentrations were approximately 100-fold higher than those of adrenaline and dopamine, reflecting both pronounced endogenous release and high-dose exogenous administration of noradrenaline, whereas adrenaline and dopamine were neither supplemented nor substantially elevated. At such low concentrations, a proportional reduction across the filter cannot be reliably detected, so the absence of a measurable gradient for adrenaline and dopamine does not by itself prove a true absence of adsorption. Second, a genuinely substance-specific component cannot be excluded. Activated charcoal adsorbs solutes predominantly through hydrophobic and π-π interactions, and differences in molecular structure, polarity, and plasma protein binding among catecholamines may modulate adsorption affinity [20,21]. In the absence of dedicated in-vitro adsorption experiments, however, this physicochemical mechanism remains hypothetical. Taken together, the observed pattern most likely reflects a combination of concentration-dependent detectability and possible substance-specific adsorption, and the relative contribution of each warrant further investigation. From a physiological standpoint, this selectivity is clinically meaningful, as it suggests that not all externally administered vasopressors are equally affected by extracorporeal filtration [22]. In our institutional practice, vasopressin is routinely administered as an adjunct to counteract peri-procedural hemodynamic instability. Clinically, this strategy has been associated with more reliable hemodynamic stabilization, as demonstrated in our previous work [10]. Further studies are therefore warranted to systematically investigate potential interactions between the filtration system and other endogenous or exogenously administered vasoactive mediators.

Our findings extend and mechanistically contextualize previous clinical observations reported in the chemosaturation literature. Several studies have consistently described significant hemodynamic instability during hepatic chemosaturation, particularly during inferior vena cava occlusion and the active filtration phase, with high vasopressor demands and frequent hypotensive episodes [10,11,23–25]. However, these reports largely attributed circulatory failure to preload reduction, systemic inflammatory responses, or the effects of ECC itself, without direct biochemical evidence of mediator loss. While their work focused on clinical management strategies and outcomes, our data provide a complementary mechanistic explanation by demonstrating that noradrenaline itself is effectively removed from the circulation during filtration. Importantly, we do not interpret noradrenaline removal as the sole mechanism. The marked reduction in venous return during balloon occlusion during which lower-body venous return reaches the heart only via the extracorporeal circuit at 500 mL/min is expected to substantially reduce cardiac preload and output and likely acts in parallel. Our data nonetheless indicate that filter-mediated noradrenaline extraction is not reducible to preload loss: at the post-filter timepoint, measured noradrenaline concentrations were lowest despite the highest infusion rates, a dissociation between administered dose and circulating concentration that preload reduction alone cannot explain. Our findings extend previous experimental animal data and case series demonstrating catecholamine depletion by activated charcoal filtration and support the hypothesis that filter-mediated catecholamine loss represents a key mechanistic contributor to the hemodynamic instability observed during hepatic chemosaturation [13,23].

After termination of ECC, noradrenaline concentrations rose approximately 4-fold the T1 and 18-fold the T0 concentration. This rebound occurred despite down-titration of exogenous noradrenaline and therefore provides indirect confirmation that active trans-filter extraction occurred during filtration. The observed rebound further underscores the dynamic interplay between endogenous stress responses, exogenous vasopressor administration, and filter-mediated adsorption. Once the charcoal filter is bypassed, ongoing endogenous catecholamine release and continued exogenous infusion may result in supraphysiological circulating levels, potentially predisposing patients to hypertension or tachyarrhythmias if vasopressor therapy is not promptly adjusted. This rebound phenomenon has important implications for post-ECC hemodynamic management and highlights the need for anticipatory rather than reactive vasopressor titration.

From a clinical perspective, our findings have several direct implications. First, anesthesiologists should anticipate substantial loss of noradrenaline during active charcoal filtration and recognize that escalating infusion rates may have limited hemodynamic benefit during this phase. Second, early use of alternative vasopressors that might be less susceptible to filter adsorption appears physiologically justified and is supported by existing clinical practice patterns reported in larger cohorts [10,12]. Third, awareness of the selective nature of catecholamine removal may inform the future development of procedure-specific hemodynamic algorithms, including proactive vasopressor combinations, careful volume management, and vigilant monitoring during transitions into and out of ECC.

Besides immediate clinical management, these findings also raise broader considerations regarding extracorporeal system design. Activated charcoal filters are optimized for chemotherapeutic adsorption but may unintentionally remove critical endogenous mediators. Non-specific adsorption by this filter was already documented by Moeslein et al., who reported that multiple blood constituents beyond melphalan (albumin, platelets, neutrophils, fibrinogen) were measurably reduced, attributed in part to filter adsorption. Notably, the same authors explicitly called for future evaluation of catecholamine extraction to better understand peri-procedural blood pressure management [26]. Future refinements in filter composition or circuit configuration may allow preservation of essential vasoactive substances while maintaining oncological efficacy.

Beyond hemodynamic alterations, hepatic chemosaturation was associated with progressive metabolic acidosis and rising lactate concentrations during and after ECC, as also described before [25]. Although the absolute lactate increase remained moderate, its temporal pattern is mechanistically informative. Several processes are likely to contribute. First, transient systemic hypoperfusion during inferior vena cava occlusion and impaired vasomotor regulation due to noradrenaline depletion may promote anaerobic lactate generation at the microcirculatory level. Second, the liver as the principal organ of lactate clearance is simultaneously subjected to regional ischemia and to complete diversion of its venous outflow during hepatic isolation, so that lactate production within the splanchnic bed coincides with a temporary loss of hepatic lactate disposal [27]. Third, direct cytotoxic effects of high-dose melphalan on hepatocellular mitochondrial function may further shift hepatic metabolism toward anaerobic glycolysis [28]. Finally, the observation that lactate peaked at T3 rather than during active filtration is consistent with washout of accumulated lactate from the previously isolated hepatosplanchnic circulation upon restoration of venous return, superimposed on still-recovering hepatic clearance. Accordingly, hyperlactatemia in this setting should be interpreted as a composite marker of regional ischemia, transiently impaired hepatic clearance and systemic perfusion mismatch rather than as evidence of global circulatory shock alone. Notably, metabolic recovery lagged behind macrocirculatory stabilization after termination of ECC, indicating that restoration of blood pressure alone may not immediately normalize cellular metabolism. These observations underscore the importance of integrating metabolic monitoring into peri-procedural management and highlight that metabolic acidosis and hyperlactatemia should be interpreted as markers of systemic stress and perfusion mismatch rather than isolated laboratory abnormalities [29,30]. Proactive strategies aimed at maintaining perfusion pressure, optimizing vasopressor selection, and avoiding excessive catecholamine rebound may help mitigate downstream metabolic consequences.

This study is limited by the assessment of systemic plasma catecholamines only, which may not reflect local tissue-level activity or receptor sensitivity. Furthermore, anesthesia and hemodynamic management were guided by institutional standards rather than a fixed study protocol. Potential variations in fluid therapy and vasopressor dosing may have influenced the circulating catecholamine levels and the resulting hemodynamic data. Also, cardiac output, systemic vascular resistance, and pulmonary artery pressures were not measured. Hemodynamic assessment relied on MAP, heart rate, CVP, PPV, and perfusion index. Additionally, this was a single-center, exploratory study with a small sample of 15 procedures and no a priori sample size calculation. While the primary effect was estimated with high precision and consistent direction across all procedures, the p-values of the secondary outcomes should be interpreted as hypothesis-generating rather than confirmatory, and the findings require confirmation in larger, multicenter cohorts. Finally, no data were collected beyond T3. The study deliberately focused on the intra-procedural mechanistic characterization of filtration-associated shock. For the post-procedural course, outcomes, and complications of chemosaturation patients at our center, we refer to our previous cohort [10].

In summary, this study identifies selective and near-complete noradrenaline adsorption by activated charcoal filtration as a key biochemical mechanism underlying circulatory failure with shock during hepatic chemosaturation. By linking catecholamine kinetics directly to hemodynamic alterations, our data provide a coherent pathophysiological framework that complements existing clinical observations and offers a rational basis for optimized peri-procedural hemodynamic management.

## Conclusion

This study provides direct biochemical evidence that activated charcoal filtration during hepatic chemosaturation selectively and near-completely removes circulating noradrenaline. This pronounced and substance-specific adsorption offers a mechanistic explanation for the severe peri-procedural hemodynamic instability and high vasopressor requirements frequently observed during ECC. Recognition of noradrenaline depletion as a key pathophysiological mechanism has direct clinical implications for peri-procedural hemodynamic management, including anticipatory vasopressor strategies and careful titration during transitions into and out of ECC with engaged activated carbon filters. These findings provide a physiological framework to optimize anesthetic management and may inform future refinements of extracorporeal filtration systems.

## Supporting information

S1 File PLOS human participants research checklist chemosatV1. (PDF)

S2 File Studienprotokoll engl. (DOCX)

S3 File Studienprotokoll engl. (PDF)

S4 File Trend chemosat. (PDF)

## Abbreviations

ACT: Activated Clotting Time
ASA: American Society of Anesthesiologists
BE: Base Excess
BMI: Body Mass Index
CAD: Coronary Artery Disease
CKD: Chronic Kidney Disease
CO: Cardiac Output
CVP: Central Venous Pressure
ECC: Extracorporeal Circulation
HR: Heart Rate
IJV: Internal Jugular Vein
IQR: Interquartile Range
IVC: Inferior Vena Cava
MAP: Mean Arterial Pressure
NA: Noradrenaline
PI: Perfusion Index
PPV: Pulse Pressure Variation
TEE: Transesophageal Echocardiography
