# Supplementary material for: Activated charcoal filtration profoundly alters systemic noradrenaline concentrations during hepatic chemosaturation: A prospective cohort study
Source: PLoS One. 2026 Aug 31;21(8):e0350771. doi: 10.1371/journal.pone.0350771 (PMC13528978; doi:10.1371/journal.pone.0350771)
Supplement: Supplementary file 2 [file pone.0350771.s003.pdf]

Klinisches Forschungsprojekt - Studienprotokoll

Vergleich der prä- und post-Filter Plasma-Katecholamin Konzentration bei Durchführung der Chemosaturation während der Nutzung des Kohlefilters

---

Beteiligte Personen

**Prüfarzt/Studienleiter:**	**Klinikdirektor**
Dr. med. Maximilian Dietrich, DESAIC<br>Facharzt<br>Klinik für Anästhesiologie<br>Universitätsklinikum Heidelberg<br>Im Neuenheimer Feld 420<br>69120 Heidelberg<br>Maximilian.dietrich@med.uni-heidelberg.de<br>+49 (0) 6221 56 38459	Prof. Dr. med. Markus A. Weigand<br>Ärztlicher Direktor<br>Klinik für Anästhesiologie<br>Universitätsklinikum Heidelberg<br>Im Neuenheimer Feld 420<br>69120 Heidelberg

Biometriker

Biometrische Beratung durch Herrn Prof. Dr. rer. nat. Kohl von der Fachhochschule Villingen-Schwenningen.

**Weitere an der Studie Beteiligte:**<br>Doktorand<br>Jan Turra, M.Sc. ECCP<br>Cardiovascular Perfusionist<br>Klinik für Herzchirurgie<br>Universitätsklinikum Heidelberg<br>Im Neuenheimer Feld 420<br>69120 Heidelberg<br>Jan.turra@med.uni-heidelberg.de<br>+49 (0) 6221 56 36118	Dr. med. Lars Werntz, M.Sc.<br>Facharzt, Apotheker<br>Klinik für Anästhesiologie<br>Universitätsklinikum Heidelberg<br>Im Neuenheimer Feld 420<br>69120 Heidelberg<br>Lars.Werntz@med.uni-heidelberg.de<br>+49 (0) 6221 56 39426
Oliver Biecker<br>Cardiovascular Perfusionist<br>Klinik für Herzchirurgie<br>Universitätsklinikum Heidelberg<br>Im Neuenheimer Feld 420<br>69120 Heidelberg<br>Oliver.Biecker@med.uni-heidelberg.de<br>+49 (0) 6221 56 36118	Prof. Dr. Dr. med. Dania Fischer<br>Oberärztin<br>Klinik für Anästhesiologie<br>Universitätsklinikum Heidelberg<br>Im Neuenheimer Feld 420<br>69120 Heidelberg<br>Dania.Fischer@med.uni-heidelberg.de<br>+49 (0) 6221 56 36166
PD Dr. med. Philipp Mayer<br>Oberarzt<br>Klinik für Radiologie<br>Universitätsklinikum Heidelberg<br>Im Neuenheimer Feld 420<br>69120 Heidelberg<br>Philipp.Mayer@med.uni-heidelberg.de<br>+49 (0) 6221 56 37345	Herr Apl. Prof. Dr. Mark O. Wielpuetz<br>Stellv. Ärztlicher Direktor<br>Klinik für Radiologie<br>Universitätsklinikum Heidelberg<br>Im Neuenheimer Feld 420<br>69120 Heidelberg<br>Mark.Wielpuetz@med.uni-heidelberg.de<br>+49 (0) 6221 56 37085
PD Dr. med. Mascha Fiedler-Kalenka<br>Oberärztin<br>Klinik für Anästhesiologie<br>Universitätsklinikum Heidelberg<br>Im Neuenheimer Feld 420<br>69120 Heidelberg<br>Mascha.fiedler-kalenka@med.uni-heidelberg.de<br>+49 (0) 6221 56 39434	Prof. Dr. Dr. med. Christoph Lichtenstern<br>Stellv. Klinikdirektor<br>Klinik für Anästhesiologie<br>Universitätsklinikum Heidelberg<br>Im Neuenheimer Feld 420<br>69120 Heidelberg<br>Christoph.lichtenstern@med.uni-heidelberg.de<br>+49 (0) 6221 56 34627

Finanzielle oder ideelle Unterstützung

Es besteht keine finanzielle oder ideelle Unterstützung der vorliegenden Studie durch andere Institutionen, Organisationen, Firmen oder Stiftungen. Die Studie wird im Rahmen einer Promotion an der Universitätsklinik Heidelberg durchgeführt.

Datum und Version

04.04.2024 / Version 1.0

Inhaltsverzeichnis

Beteiligte Personen 1

Biometriker 1

Finanzielle oder ideelle Unterstützung 3

Datum und Version 3

04.04.2024 / Version 1.0 3

Abbildungsverzeichnis 5

Abkürzungsverzeichnis 6

1 Wissenschaftlicher Hintergrund 7

2 Ziel der Studie 9

2.1 Hauptzielkriterien 10

2.2 Nebenzielkriterien 10

2.3 Hypothese 11

3 Prüfverfahren 11

3.1 Allgemeine Beschreibung 11

3.2 Unerwünschte Wirkungen, Risiken und Belastungen für den Studienteilnehmer 11

3.3 Unerwünschte Ergebnisse 11

4 Studiendesign(-typ) 12

5 Einwilligungsfähigkeit und Einschlusskriterien 12

6 Ausschlusskriterien 12

7 Randomisierungsverfahren .... **Fehler! Textmarke nicht definiert.**

8 Studienablauf 13

8.1 Studienbedingte Maßnahmen 13

8.2 Beteiligte Kliniken 13

8.3 Patientenkollektiv 14

8.4 Durchführung der Studie 14

8.4.1 Bestimmung der Serumkatecholaminspiegel 14

(a) Erhebung der allgemeinen Anamnesedaten....... **Fehler! Textmarke nicht definiert.**

(b) Erhebung intraoperativer und postoperativer Verlaufsparameter**Fehler! Textmarke nicht definiert.**

(c) Erhebung klinischer Basisdaten sowie klinisch-chemischer Parameter 15

(d) Erhebung von Verlaufsparametern und Komplikationen**Fehler! Textmarke nicht definiert.**

- Krankenhausaufenthalt, Dauer der Intensivtherapie**Fehler! Textmarke nicht definiert.**

8.4.2 Begleittherapie ....**Fehler! Textmarke nicht definiert.**

8.5 Abbruchkriterien 16

9 Statistisches Design 17

10 Rechtliche und Ethische Aspekte 17

10.1 Datenschutz 18

10.2 Meldung beim Regierungspräsidium ....**Fehler! Textmarke nicht definiert.**

10.3 Patientenversicherung 18

10.4 Patienteneinwilligung 18

10.5 Freiwilligkeit der Teilnahme und Rücktritt 18

10.6 Aufklärung und Einwilligung 19

10.7 Wirken und Nutzen 19

10.8 Finanzierung, Interessenkonflikt und Patientenrisiko 19

11 Datenerhebungsbögen 20

Literaturverzeichnis ....**Fehler! Textmarke nicht definiert.**

Unterschriftenseite 22

Anlage 1 23

Abbildungsverzeichnis

Abbildung 1: Ein schematischer Aufbau des Hepatic CHEMOSAT Delivery 9

Abkürzungsverzeichnis

EKZ	Extrakorporale Zirkulation
h	Stunde
MAD	mittlerer arterieller Druck
NO	Stickstoffmonoxid
RA	rechte Atrium
VCI	V. cava inferior
VJI	V. jugularis interna

1 Background

The Hepatic CHEMOSAT Delivery System (Delcath Systems Inc., New York, NY, USA) represents an innovative therapeutic option for inoperable primary liver tumors and liver metastases aimed at palliative symptom management and the prolongation of survival. This procedure involves applying a high dose of the chemotherapeutic agent melphalan directly into the liver while simultaneously keeping systemic exposure for the entire organism as low as possible. Although the original concept of this procedure was developed decades ago, it has not yet established itself in routine clinical practice [1], [2]. On April 13, 2011, the Hepatic CHEMOSAT Delivery System received CE (Conformité Européenne) certification from the European Union (EU) and has been in commercial use in the EU since 2012.

- Indications: The procedure can be used for patients with primary liver tumors, such as hepatocellular or cholangiocellular tumors, as well as for liver metastases.
- Local Use: In Heidelberg, this procedure has been used since 2016 primarily for inoperable, central liver metastases, particularly those originating from uveal melanomas [3].

The Hepatic CHEMOSAT Delivery System consists of various sterile, single-use materials, including:

- Intravascular catheters.
- An extracorporeal circuit (ECC) with charcoal filters for hemofiltration.

These materials are provided anew by Delcath Systems Inc. for every intervention. The extracorporeal circulation is provided by the Department of Cardiovascular Technology (Clinic for Cardiac Surgery) and is operated by a perfusionist during the procedure. The required chemotherapeutic agent, melphalan hydrochloride, is prepared individually for each patient in the hospital's internal pharmacy.

Melphalan is considered an ideal chemotherapeutic agent for this application because it has demonstrated high efficacy in treating primary liver tumors and a variety of liver metastases of different origins, while also possessing reversible liver toxicity [4]–[8].

The primary systemic toxicity of melphalan consists of myelosuppression, which can include:

- Thrombocytopenia
- Anemia
- Neutropenia [9]

The chemosaturation is performed in an angiography suite under general anesthesia and lasts approximately 3–4 hours.

1. Administration: The chemotherapeutic agent is applied directly into the tumor-supplying arterial branches of the hepatic artery via a catheter placed in the left femoral artery.
2. Isolation: A double-balloon catheter is inserted via the left femoral vein.
    - The upper balloon is positioned at the junction of the inferior vena cava (IVC) and the right atrium (RA).
    - The lower balloon is placed infrahepatically in the IVC, isolating the venous outflow from the hepatic veins between the two balloons.
3. Filtration: The ECC system suctions the venous, toxic hepatic blood—fully saturated with the chemotherapeutic agent—through the double-balloon catheter. The blood passes through two charcoal filters, which filter the chemotherapeutic agent out of the blood.
4. Reinfusion: The purified blood is then returned to the patient’s systemic circulation via a sheath in the right internal jugular vein (IJV).

Note on Hemodynamics: Due to reactions within the charcoal filter, this filtration phase of the procedure leads to massive drops in blood pressure (BP drop of > 100 mmHg) and significant hemodynamic instability [10]

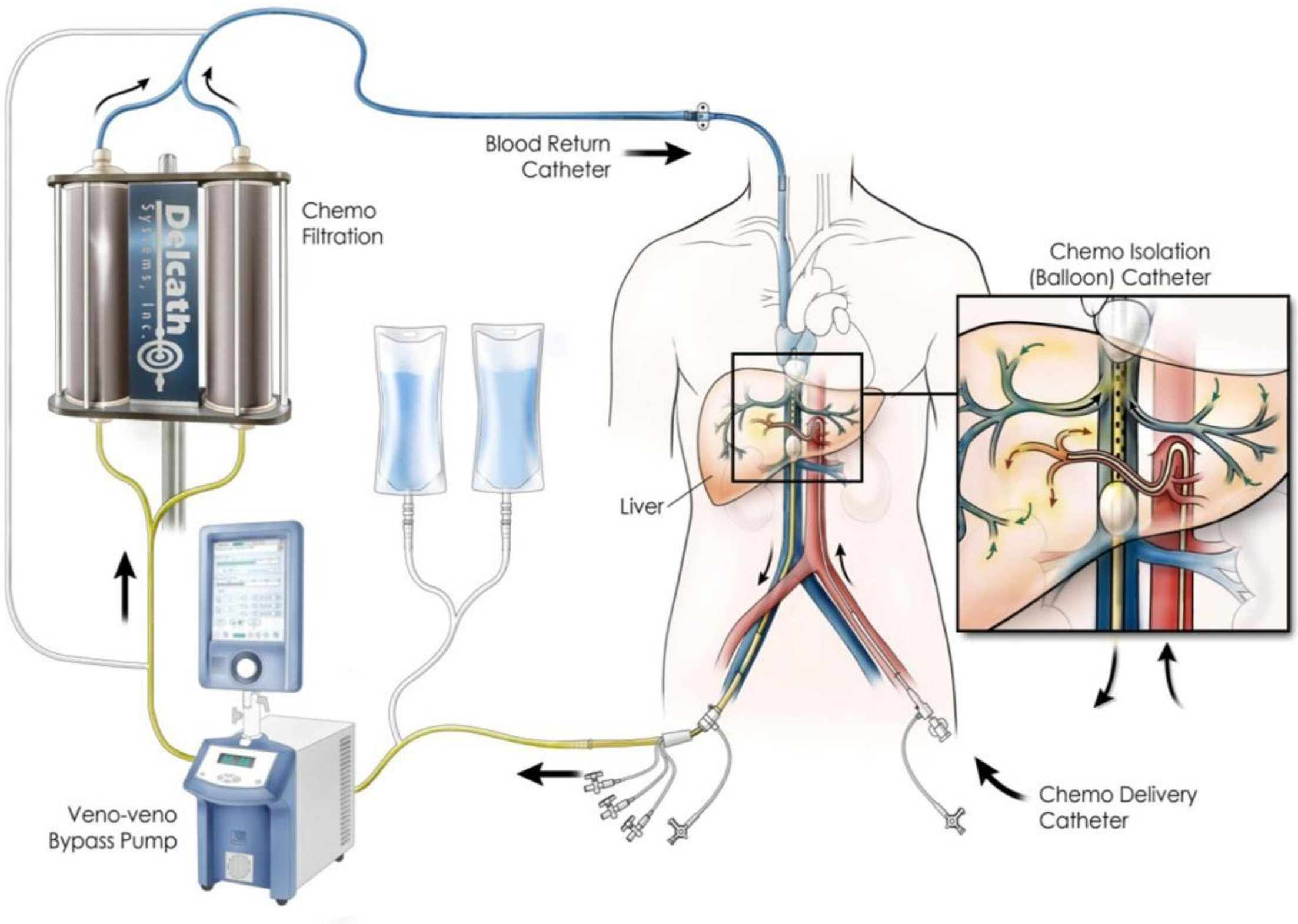

Figure 1: Hepatic CHEMOSAT Delivery System

Due to the extreme hemodynamic fluctuations during the procedure, it is considered a high-risk intervention from an anesthesiological perspective.

The anesthesiological monitoring is correspondingly complex and includes:

- 5-lead ECG monitoring.
- Pulse oximetry.
- Non-invasive and invasive blood pressure measurement.
- A central venous catheter (CVC) with continuous central venous pressure measurement.
- An indwelling urinary catheter.
- Since 2019: Intraoperative transesophageal echocardiography (TEE).

Following the induction of anesthesia, the return venous catheter of the Hepatic CHEMOSAT Delivery System is placed into the right internal jugular vein (IJV) by the anesthesia team, along with the other invasive equipment described. The remaining required arterial and venous catheters, including the double-balloon catheter, are established by the radiology department.

A massive circulatory failure occurs upon the initiation of the charcoal filter. Currently, there are no studies in the literature investigating its causes. Instead, various theories are discussed, which assume one of the following:

- Catecholamine filtration by the system.
- A systemic inflammatory response with cytokine release.
- The release or upregulated synthesis of nitric oxide (NO) [2].

We intend to investigate these possibilities in this study. Additional insights into the pathomechanism of circulatory failure gained through the planned measurements of intraoperative catecholamine levels could be utilized to improve intraoperative hemodynamic therapy.

2 Study Goals

The objective is to measure catecholamine levels before and after the charcoal filter at various intraoperative time points and subsequently determine whether the charcoal filter filters catecholamines out of the patient's blood during chemosaturation.

2.1 Main Goals

- Laboratory-diagnostic measurement of catecholamine levels during chemosaturation (pre- and post-filter)

2.2 Other Goals

- Assessment of the correlation between the duration of inferior vena cava occlusion and catecholamine levels.
- Nitric oxide (NO) measurement via the existing mechanical ventilation.
- Measurement of inflammatory markers.
- Correlation of catecholamine levels with hemodynamic variables (blood pressure, cardiac output, heart rate).
- Correlation of blood catecholamine levels with hemodynamic therapy (vasopressor/fluid administration).

2.3 Hypothesis

- H1-Hypothesis
  Activating the charcoal filter in the ECC circuit reduces the catecholamine levels in the blood.
- 0 Hypothesis
  Activating the charcoal filter in the ECC circuit leaves the catecholamine levels unchanged.

3 Study Design

3.1 General Description

This is a prospective, monocentric clinical study. The chemosaturation is performed according to the established standard. Within the scope of this study, only several serial blood samples are taken

3.2 Adverse Effects, Risks, and Burdens for Study Participants

For this prospective, monocentric clinical study, six blood samples using one serum tube each are required. Each tube requires 2.7 ml of whole blood, resulting in a total volume of 16.2 ml.

The volume of whole blood required is so minimal that it poses no additional burden to the patients. Furthermore, the blood samples are taken from indwelling catheters already required for the procedure or directly from the ECC (extracorporeal circulation); therefore, no additional punctures or other invasive measures are necessary for the study. Additionally, data routinely collected during standard chemosaturation will be evaluated.

3.3 Adverse Events

Based on the following explanations, no additional risk to the patient during surgery is expected:

- The standardized ECC procedure is used as in any regular chemosaturation.
- There are no modifications to the single-use system and no changes to the radiological or anesthesiological strategy.
- Planned blood samples are taken via connected three-way stopcocks.

- Samples are analyzed via the central laboratory.

4 Study Design (Type)

The presented investigation is a prospective, monocentric clinical study. It is therefore not an AMG study (Medicinal Products Act), a non-interventional study (observational), or an MPDG study (Medical Devices Law Implementation Act).

5 Consent Capacity and Inclusion Criteria

The investigations are conducted at the University Hospital Heidelberg in the Department of Radiology. Patient gender is irrelevant to this study. Only adults capable of giving informed consent will be included.

- Informed Consent: Information and consent procedures take place during the anesthesiological premedication visit.
- Data Collection: With the exception of blood tests, only patient data routinely collected during the hospital stay will be recorded.
- Target Enrollment: The goal is to include 40 patients over a period of 24 months.

Inclusion Criteria:

- Age > 18 years
- Elective chemosaturation with ECC

6 Exclusion Criteria

Patient Refusal: Denial of consent by the patient to participate in the study.

Pheochromocytoma: Patients with a known pheochromocytoma (adrenal tumor) are excluded. As a hormonally active tumor, it secretes catecholamines, which would invalidate the measurement results.

.

8 Study Procedure

8.1 Study related measures

The patient is brought to the induction room, where they are prepared and equipped with all catheters intended for the procedure.

Sampling Timeline:

- T0: The first blood sample is taken following the successful induction of anesthesia.
- T1: After connecting the ECC (Extracorporeal Circulation) to both catheters, blood from the liver is initially pumped via a bypass—avoiding the charcoal filters—back into the jugular vein using a centrifugal pump. Once the target blood flow of 500 ml/min is reached and the patient is hemodynamically stable, the second sample is taken.
- T2 and T3 : Following successful occlusion of the balloon catheter, the bypass line is clamped, and the clamps for the charcoal filters are opened. The filters are flooded with blood one after the other. Once the patient's circulation is stable and the radiologist is ready to begin the application of the chemotherapeutic agent, samples are taken pre-filter () and post-filter () (prior to chemo administration).
- T4: During the final phase of chemosaturation, the venous blood from the liver is filtered. This washout phase lasts for an additional 30 minutes. Afterward, the balloons of the isolation catheter are opened sequentially. The next blood sample is taken immediately before the charcoal filter is clamped.
- T5: The ECC is reduced and terminated, and the remaining blood in the system is returned to the patient. The final blood sample is taken in the angiography suite prior to emergence from anesthesia.

8.2 Clincs

Klinik für Anästhesiologie

Universitätsklinikum Heidelberg

Im Neuenheimer Feld 420

69120 Heidelberg

Klinik für Diagnostische und Interventionelle Radiologie Klinik für Radiologie

Universitätsklinikum Heidelberg

Im Neuenheimer Feld 420

69120 Heidelberg

Klinik für Herzchirurgie

Abteilung für Kardiotechnik

Im Neuenheimer Feld 420

69120 Heidelberg

8.3 Patients

Patients included in this study are those undergoing a radiological intervention for chemosaturation via ECC at the University Hospital Heidelberg. The inclusion criteria have been detailed in the previous sections. It is anticipated that a total of 40 patients will be examined; further justification for this sample size is provided in Section 9.

8.4 Study Implementation

8.4.1 Determination of Serum Catecholamine Levels

Catecholamine levels will be recorded at the following six measurement time points:

- T0: Baseline; after induction of anesthesia.
- T1: During ECC without the charcoal filter in the system.
- T2 and T3: Pre-filter and post-filter, with the filter active in the system, prior to administration of the chemotherapeutic agent.
- T4: Shortly before the end of ECC, post-filter.
- T5: After the end of ECC; prior to emergence from anesthesia.

Procedure: At the designated collection points, a serum tube is filled with 2.7 ml of whole blood, subsequently centrifuged, and sent to the laboratory for analysis.

.

(a) General medical history

The following data will be collected:

- Age, sex, weight, height, body surface area (BSA), ASA classification.
- Current long-term medication.
- Pre-existing conditions: NYHA class, history of myocardial infarction, coronary function, echocardiography findings, physical exercise capacity, neurology, liver/lung/kidney function, hypertension/hypotension, vascular diseases, diabetes, other metabolic disorders, oncological history, etc.
- Other history: Allergies, alcohol and nicotine consumption, substance abuse, etc.
- Previous surgeries.

(b) Intraoperative and Postoperative Parameters

Parameters are gathered from anesthesia records and ICU documentation systems. These data are generated as part of routine clinical care:

- Ventilation: FiO_2, PEEP, respiratory rate, ventilation mode, .
- Arterial Blood Gas (ABG): pH, pO_2, pCO_2, SaO_2, HCO_3, Base Excess (BE), lactate.
- Central Venous Blood Gas (CVBG): see ABG
- Hemodynamics: Arterial pressure (systolic/diastolic min/max), MAP (min/max), CVP (min/max), Heart Rate (min/max).
- Fluid Balance: * Intake: Crystalloids, colloids (e.g., human albumin), blood products (PRBC, FFP, platelets).
    - Output: Urine, blood loss, other losses.
- Temperature and catecholamine therapy.

(c) Clinical Basic Data and Clinico-chemical Parameters

These are recorded during the hospital stay as part of routine care. **No study-related blood draws or examinations will be performed for these parameters!**

- **General Infectiology (pre- and post-intervention):** Leukocytes, CRP, PCT, temperature (min/max).

- **Routine Lab:** Red blood cell count, hemoglobin, hematocrit, platelets, coagulation (Quick/INR, PTT, ATIII, fibrinogen, Protein C/S, Factor XIII, D-dimers), creatinine, urea, blood glucose, liver enzymes (GOT, GPT, GGT, CHE, AP, LDH), bilirubin (total/direct), lactate, ammonia, amylase, lipase, total protein, albumin, electrolytes (Na, K, Ca, Mg, Cl), cardiac markers (TnT, TnI, CK, CK-MB, BNP).
- **Scores:** APACHE II, SAPS, SOFA.
- **Care Data:** Duration of ventilation and overall treatment.

(d) Follow-up Parameters and Complications

- Duration of hospital stay and intensive care treatment.
- **Organ Systems:** Acute Kidney Injury (AKI), ARDS, Acute Liver Failure (ALF).
- Mortality and causes of death.
- Subsequent surgeries and interventions.
- General complications.

8.5 Discontinuation

Medical adverse events for patients are not expected as a result of the study, as it is an observational study without intervention or influence on patient care. The study will be discontinued if it is methodologically impossible to measure catecholamines in the laboratory (e.g., due to interference between melphalan and the laboratory measurement method).

9 Statistics

A concrete sample size estimation cannot be performed due to the pilot nature of the study. Data collection for this project will be conducted using an electronic database system (Microsoft Excel). Subsequently, statistical analysis will be performed using SPSS (Statistical Product and Services Solutions).

- Descriptive Statistics: A detailed descriptive analysis of all collected data will be conducted.
- Univariate Statistics: * T-test for normally distributed continuous variables/scores.
    - Mann-Whitney test for non-normally distributed continuous variables/scores.
    - Chi-square test for the evaluation of categorical variables.
- Long-term Evaluation: Mortality and the occurrence of complications within 28, 90, and 180 days will be analyzed using Kaplan-Meier analysis. For this purpose, patients will be interviewed during follow-up examinations in the Radiology Department.
- Multivariable Methods: Logistic regression models for morbidities and Cox regression models for mortality will serve to evaluate potential prognostic factors.
- Significance: Results are to be interpreted as purely descriptive and have no confirmatory value. The significance level is set at 5%.

10 Legal and Ethical Aspects

The investigation will be conducted in accordance with the Declaration of Helsinki and the Professional Code of Conduct for Physicians of the Baden-Württemberg Medical Association in their currently applicable versions [12].

This is a prospective, monocentric clinical study investigating whether the charcoal filter removes catecholamines during chemosaturation, thereby leading to an increased requirement for norepinephrine administration to stabilize the mean arterial pressure.

The study protocol will be submitted to the Ethics Committee of the Heidelberg Medical Faculty for review prior to the start of the study. Patient enrollment will not begin until a written, favorable opinion from the Ethics Committee has been received.

Patient names and all other confidential information are subject to medical confidentiality and the provisions of the Federal Data Protection Act (BDSG), the General Data Protection Regulation (GDPR), and the State Data Protection Act (LDSG). Patient data will only be shared in anonymized form. Third parties will not be granted access to original medical records.

10.1 Data Protection

All data identifying the patient are accessible only to the study physicians. For all other persons involved in the study, the data will be pseudonymized and encrypted. Only anonymized patient data will be used in publications.

- Access: The key linking patient codes to patient names is held exclusively by the physicians from the Department of Anesthesiology involved in the study.
- Decoding: Decrypting pseudonymized data is only permissible in justified cases, such as when important new findings regarding the diagnosis or therapy of the underlying disease emerge during the study.
- Archiving: For legal reasons, data are expected to be stored in the archives of the University Hospital of Surgery for 10 years.

10.3 Insurance

The project is covered by the professional liability insurance of the Heidelberg University Hospital. This covers potential damages sustained as a result of participation in the scientific study.

10.4 Consent

The provision of information and the obtaining of consent for the study shall take place during the preoperative anesthesiological briefing.

10.5 Voluntariness of Participation and Withdrawal

Participation in the investigation is strictly voluntary. Consent can be withdrawn at any time without providing reasons and without any disadvantages for further medical care.

- **Informed Consent:** Participants (or their legal representatives for patients lacking capacity) will be informed both orally and in writing about the nature and scope of the planned investigation, particularly regarding potential health benefits and risks.
- **Risk/Benefit:** As this is an observational study, it involves only minimal burden or risk. Participants derive no direct personal benefit from their participation.
- **Refusal:** Any refusal to participate by the subject, provided they are capable of verbalizing their will, will be respected at all times (Articles 28–30 of the Declaration of Helsinki).
- **Documentation:** Consent is documented by signature on the informed consent form.
- **Data Management upon Withdrawal:** If a participant withdraws, any (data) material already obtained will be destroyed, or the patient (or legal representative) will be asked whether they agree to the continued evaluation of the material (only possible if pseudonymized).

10.6 Information and Consent

Participants or their legal representatives will be informed orally and in writing about the nature and scope of the investigation. As this is an observational study, there are only minimal burdens or risks. There is no direct benefit to the participants.

10.7 Impact and Benefit

While study participants do not benefit personally, the study may lead to an improvement in care for potential future procedures.

10.8 Financing, Conflict of Interest, and Patient Risk

Conflicts of Interest: There are no institutional or financial conflicts of interest for the responsible parties involved in this study.

Financing: Since the study utilizes normal clinical operations, no external funding is required.

Patient Risk: Participation involves no additional risk beyond that of the actual procedure itself, as the study entails no changes to therapy or protocol. The small volume of blood drawn for the study is not expected to cause any relevant additional burden.

11 Datenerhebungsbögen

All collected laboratory parameters and clinical baseline data will be stored in an Excel spreadsheet.

References

[1] Y. Ku *et al.*, “Reductive surgery plus percutaneous isolated hepatic perfusion for multiple advanced hepatocellular carcinoma.,” *Ann. Surg.*, vol. 239, no. 1, pp. 53–60, Jan. 2004.

[2] A. Vogel *et al.*, “Chemosaturation Percutaneous Hepatic Perfusion: A Systematic Review.,” *Adv. Ther.*, vol. 33, no. 12, pp. 2122–2138, Jan. 2017.

[3] P. F. Ferrucci, E. Cocorocchio, G. Bonomo, G. M. Varano, P. Della Vigna, and F. Orsi, “A New Option for the Treatment of Intrahepatic Cholangiocarcinoma: Percutaneous Hepatic Perfusion with CHEMOSAT Delivery System,” *Cells*, vol. 10, no. 1, p. 70, Jan. 2021.

[4] A. Marinelli, A. L. Vahrmeijer, and C. J. van de Velde, “Phase I/II studies of isolated hepatic perfusion with mitomycin C or melphalan in patients with colorectal cancer hepatic metastases.,” *Recent Results Cancer Res.*, vol. 147, pp. 83–94, 1998.

[5] A. L. Vahrmeijer *et al.*, “Increased local cytostatic drug exposure by isolated hepatic perfusion: a phase I clinical and pharmacologic evaluation of treatment with high dose melphalan in patients with colorectal cancer confined to the liver.,” *Br. J. Cancer*, vol. 82, no. 9, pp. 1539–46, May 2000.

[6] M. R. de Vries *et al.*, “Isolated hepatic perfusion with tumor necrosis factor alpha and melphalan: experimental studies in pigs and phase I data from humans.,” *Recent Results Cancer Res.*, vol. 147, pp. 107–19, 1998.

[7] L. B. J. van Iersel *et al.*, “Isolated hepatic perfusion with oxaliplatin combined with 100 mg melphalan in patients with metastases confined to the liver: A phase I study.,” *Eur. J. Surg. Oncol.*, vol. 40, no. 11, pp. 1557–63, Nov. 2014.

[8] L. B. J. van Iersel *et al.*, “Isolated hepatic melphalan perfusion of colorectal liver metastases: outcome and prognostic factors in 154 patients.,” *Ann. Oncol. Off. J. Eur. Soc. Med. Oncol.*, vol. 19, no. 6, pp. 1127–34, Jun. 2008.

[9] W. Jung, F. Zettl, R. Schroers, K. Wolfram, and J. · Georg, “Glossar DSMM = Deutsche Studiengruppe Multiples Myelom GMMG = German-speaking Myeloma Multicenter Group OSHO = Ostdeutsche Studiengruppe Hämatologie/Onkologie TRM = Therapie-bezogene Mortalität VAD = Vincristin, Doxorubicin, Dexamethason VID = Vincristin,.”

[10] H. Keller, G. Lotz, S. Zangos, M. Meissner, and T. Vogl, “Extrakorporale Perfusion mit dem Delcath-System: Methodik der intraarteriellen ultrahochdosierten Chemo- therapie der isolierten Leber,” *Kardiotechnik*, vol. 2, pp. 37–40, 2014.

[11] O. Martin-Delgado *et al.*, “Percutaneous Isolated Hepatic Perfusion: Hemodynamic Monitoring and Goal-Directed Anesthetic Management: A Case Report.,” *A A case reports*, vol. 8, no. 11, pp. 300–303, Jun. 2017.

[12] Weltärztebund (WMA), “WMA Deklaration von Helsinki -Ethische Grundsätze für die medizinische Forschung am Menschen,” *WMA-Generalversammlung WMA-Generalversammlung WMA-Generalversammlung*, vol. 35, no. 53, 1964.

Unterschriftenseite

Beteiligte Institute

Klinik für Anästhesiologie
(Ärztlicher Direktor: Prof. Dr. M.A. Weigand)
Universitätsklinikum Heidelberg
Im Neuenheimer Feld 420
69120 Heidelberg

Klinik für Herzchirurgie
(Ärztl. Direktor: Prof. Dr. M. Karck)
Universitätsklinikum Heidelberg
Im Neuenheimer Feld 420
69120 Heidelberg

Klinik für Radiologie
(Ärztlicher Direktor: Prof. Dr. med. H.-U. Kauczor))
Universitätsklinikum Heidelberg
Im Neuenheimer Feld 420
69120 Heidelberg

Beteiligte Untersucher

Prüfarzt/Studienleiter:
Dr. med. Maximilian Dietrich DESAIC
Facharzt
Klinik für Anästhesiologie
Universitätsklinikum Heidelberg
Im Neuenheimer Feld 420
69120 Heidelberg

Perfusionist /Doktorant:
Jan Turra, M.Sc., ECCP
Cardiovascular Perfusionist
Klinik für Herzchirurgie
Universitätsklinikum Heidelberg
Im Neuenheimer Feld 420
69120 Heidelberg

Unterschriften

Datum: 04.04.2024

Dr. med. Maximilian Dietrich

Datum: 04.04.2024

Jan Turra, M.Sc. ECCP

Anlage 1

Studienprotokoll

Checkliste

	Erledigt
Präoperative Datenerhebung (Patenteninfos)	
Intraoperative Datenerhebung (Vorgehensweise)	
6 Plasmaröhrchen bereit gestellt	

Präoperative Datenerhebung

Präoperative Daten	
Studienprotokollnummer	von 40
OP-Datum	
Diagnose	
Nebendiagnose	
Geschlecht	
Größe (cm)	
Gewicht (kg)	
Chemosaturationsset	
Kohlefilter	
T0 Baseline Blutentnahme nach Narkoseeinleitung	

Intraoperative Blutentnahme für die spätere Auswertung

T1	An der EKZ ohne Kohlefilter im System
T2, T3	Vor- und hinter dem Kohlefilter, wenn dieser ins System eingeschaltet wird
T4	Kurz vor Ende der EKZ hinter dem Kohlefilter

Postoperative Blutentnahme für die spätere Auswertung

T5	Nach Ende der EKZ vor Narkoseausleitung

Erklärung

Hiermit erkläre ich, Jan Turra_die Richtigkeit aller erhobenen Patienten- und Messdaten.

____

Unterschrift

Heidelberg, den
