# Supplementary material for: Activated charcoal filtration profoundly alters systemic noradrenaline concentrations during hepatic chemosaturation: A prospective cohort study
Source: PLoS One. 2026 Aug 31;21(8):e0350771. doi: 10.1371/journal.pone.0350771 (PMC13528978; doi:10.1371/journal.pone.0350771)
Supplement: Supplementary file 5 [file pone.0350771.s006.docx]

5. Review Comments to the Author

Reviewer #1: This is a prospective, single-center observational study in which systemic catecholamine concentrations were measured in fifteen procedures undergoing hepatic chemo saturation. The objective appears to be determining if the activated charcoal filter used during chemo saturation absorb catecholamines leading to profound hemodynamic instability?
The primary outcome was the within-procedure change in catecholamine concentrations across the activated charcoal filter, quantified by paired pre-filter and post-filter plasma levels of noradrenaline, adrenaline, and dopamine during ECC (times T2.1 vs. T2.2).
There were five secondary outcomes.

*🡪 We thank Reviewer 1 for the careful and encouraging assessment of our manuscript and for confirming the appropriateness of the statistical approach. We have addressed all three points and provide additional analyses where requested.*

1. Please determine if multiple comparison adjustments to the p-values for the multiple secondary outcomes are needed. All tables appear to be within any adjustment needed.

*🡪 We thank the Reviewer for raising this important point. Our study was designed as a prospective, exploratory cohort study. The primary outcome was prespecified as the within-procedure trans-filter change in catecholamine concentrations (T2.1 vs. T2.2), whereas the secondary outcomes were hypothesis-generating. In line with established methodological recommendations for exploratory studies (Bender & Lange, J Clin Epidemiol 2001), a formal adjustment for multiplicity is therefore not mandatory, provided the exploratory nature is clearly stated. We have now made this explicit in the Statistical Methods and Limitations sections.*

*To nonetheless demonstrate the robustness of our findings, we additionally applied Bonferroni, Holm and Benjamini-Hochberg (false discovery rate) corrections to all primary and secondary tests. None of our conclusions changed: the trans-filter reduction of noradrenaline remained highly significant (Holm-adjusted p = 1.8 × 10⁻⁴), as did the noradrenaline time course (Holm-adjusted p = 4.0 × 10⁻⁸) and all eleven hemodynamic and metabolic secondary outcomes (all Holm-adjusted p < 0.008). The non-significant results for adrenaline and dopamine (both trans-filter and over time) were non-significant before and after adjustment.*

2. There was no motivation for sample size , this being an observational study. However, it would have been helpful to determine the adequacy of the sample size withing margin of error expectations as is not uncommon for this type of research and since inferential p-values are presented. They included 15 chemo saturation procedures in 12 individual patients in the analysis.

*🡪We thank the Reviewer for this comment. As an exploratory observational study, no a priori sample size calculation was performed. Instead, we consecutively included all eligible chemosaturation procedures performed during the predefined study period. We have now stated this explicitly in the Methods.*

*Regarding the adequacy of the sample size: Across the 15 procedures, the trans-filter noradrenaline extraction rate was highly consistent (median 95.5%, distribution-free 95% CI 93.6–98.1%), and a reduction was observed in every single procedure (15/15), with a minimum extraction of 90.8%. As an additional precision estimate, the mean extraction rate was 95.6% (95% CI 94.3–97.0%), corresponding to a margin of error of ±1.3 percentage points. This indicates that, for the primary outcome, the sample of 15 procedures yields a precise and stable estimate. Even a sign test, which disregards effect magnitude entirely, yields p = 6.1 × 10⁻⁵ based on this concordance alone. This indicates that, for the primary outcome, the sample of 15 procedures provides a precise and stable estimate. By contrast, adrenaline (5 decreased / 5 increased / 5 unchanged) and dopamine (7/6/2) showed no directional effect, confirming that their non-significant results reflect a true absence of absorption rather than insufficient power. We have added a sentence summarizing the precision of the primary estimate to the Results and addressed the absence of an a priori power calculation in the Limitations.*

The statistical analysis is routine for the small sample consisting of continuous variables e reported as non parametric median and interquartile range (IQR). Categorical variables are reported as absolute and relative frequencies. Differences in catecholamine levels before and after filtration were analyzed using inferential non-parametric methods. For comparisons across multiple time points, the Friedman test was applied. For pairwise comparisons between pre- and post-filtration measurements, the Wilcoxon signed-rank test was used. To estimate the magnitude of the paired differences, the Hodges–Lehmann estimator with corresponding 95% confidence intervals (CI) was calculated. All the analyses were appropriate and well applied.
The paper was well written from a statistical perspective and the tables and figures were well formatted and interpretable.

The limitations section is incomplete.
3. Since the sample is so small a warning should be included with the limitations that this study is more observational with small sample size. Thus, the p-values should be interpreted with caution.

*🡪 We agree and have expanded the Limitations section to emphasize the small, single-center sample, the exploratory design, the absence of an a-priori sample size calculation, and the resulting need to interpret the p-values of the secondary outcomes as hypothesis-generating.*

Reviewer #2: The manuscript addresses a clinically relevant question related to hemodynamic instability during hepatic chemosaturation. The topic is of interest, as it attempts to provide a mechanistic explanation for a well-recognized clinical phenomenon. The study is generally well organized and based on prospective data collection.

*🡪 We thank Reviewer 2 for the positive and constructive evaluation of our manuscript and for recognizing the clinical relevance of the research question and the value of our prospective data collection. We appreciate the helpful suggestions regarding the sample size justification, the mechanistic interpretation, and several formal points. We have addressed all comments in detail below and revised the manuscript accordingly.*

The manuscript does not provide a justification for the sample size. It appears that this study was conducted as an exploratory observational cohort without a formal sample size calculation. The authors should clarify whether a priori power analysis was performed. If not, this should be explicitly stated, and the exploratory nature of the study should be emphasized.

*🡪 We thank the Reviewer for this comment. The Reviewer is correct: this was a prospective, exploratory observational cohort study, and no a priori power analysis or formal sample size calculation was performed. Instead, we consecutively included all eligible chemosaturation procedures performed at our center during the predefined study period. We have now stated this explicitly in the Methods and have emphasized the exploratory nature of the study in both the Methods and the Limitations sections.*

*To address the adequacy of the available sample, we additionally evaluated the precision of the primary effect estimate. Across the 15 procedures, the trans-filter noradrenaline extraction rate was highly consistent (median 95.5%, distribution-free 95% CI 93.6–98.1%), and a reduction was observed in every single procedure (15/15), with a minimum extraction of 90.8%. As an additional precision estimate, the mean extraction rate was 95.6% (95% CI 94.3–97.0%), corresponding to a margin of error of ±1.3 percentage points. This indicates that, for the primary outcome, the sample of 15 procedures yields a precise and stable estimate. By contrast, adrenaline and dopamine showed no directional trans-filter change, confirming that their non-significant results reflect a true absence of adsorption rather than insufficient statistical power. We have added a sentence summarizing the precision of the primary estimate to the Results, and we now explicitly address the absence of an a priori power calculation, the small single-center sample, and the resulting exploratory interpretation of the p-values in the Limitations section. We note that this point was also raised by Reviewer 1. The corresponding manuscript changes address both comments.*

Please provide a detailed explanation of the mechanism. The possible reasons why it only affects the concentration of norepinephrine need to be further elaborated.

*🡪 We thank the Reviewer for this important comment. We have substantially expanded the corresponding section of the Discussion and now offer two complementary explanations.*

*First, the apparent selectivity is to a relevant degree a consequence of the markedly different baseline concentrations of the three catecholamines. Pre-filter noradrenaline concentrations were approximately 100-fold higher (median 27,404 pmol/L) than those of adrenaline (median 274 pmol/L) and dopamine (median 448 pmol/L). This reflects the clinical setting, in which noradrenaline is both endogenously released under procedural stress and exogenously administered in high doses, whereas adrenaline and dopamine are neither supplemented nor substantially elevated. Throughout the procedure, adrenaline and dopamine concentrations remained in the low physiological range, where a proportional reduction across the filter cannot be reliably detected. Consequently, the pronounced trans-filter gradient observed for noradrenaline, and its absence for adrenaline and dopamine, is at least partly attributable to these differences in circulating concentration rather than to an exclusively substance-specific affinity of the activated charcoal. We now state this explicitly. Second, beyond this concentration effect, a genuinely substance-specific component cannot be excluded. Activated charcoal adsorbs solutes primarily through hydrophobic and π-π interactions, and small differences in molecular structure, polarity, and plasma protein binding among the three catecholamines may modulate their respective adsorption affinity. However, in the absence of dedicated in-vitro adsorption experiments, this physicochemical mechanism remains hypothetical, and we have framed it accordingly.*

*We believe that presenting both the concentration-dependent and the potential physicochemical contributions provides a more accurate and balanced mechanistic interpretation than attributing the effect to filter selectivity alone.*

Several minor language and formatting issues should be addressed. For example, an incorrect date is reported (“31.11.2025”), and there are inconsistencies in terminology (noradrenaline vs. norepinephrine). Additionally, minor formatting issues such as missing spaces between values and units (e.g., “16mmHg”) and incorrect confidence interval ordering should be corrected. These issues do not affect the scientific content but should be revised for clarity and accuracy.

*🡪 Thank you for this valuable comment. We now eliminated all inconsistencies and language errors.*

Reviewer #3: Dear authors,
here you receive my comment regarding the manuscript with the title “Activated charcoal filtration profoundly alters systemic noradrenaline concentrations during hepatic chemosaturation: a prospective cohort study”. With number PONE-D-26-16601.
The authors present their results of clinical trial. The study was approved by the local medical ethical committee with the number provided and the study was registered with the German Clinical Trials Registry.
The article is well described and the question” Does the activated charcoal filter used during chemosaturation absorb catecholamines leading to profound hemodynamic instability? ”is studied and answered.
The study is presented straight forward and easy to understand. However, I have some remarks, that maybe after answering or leading to adaptions my improve further understanding.

*🡪 We thank Reviewer 3 for the thorough and constructive evaluation of our manuscript and for the many detailed questions, which have helped us clarify the procedure and strengthen the mechanistic interpretation. We have addressed each point below and revised the manuscript accordingly.*

PGE 5 Line 105-114 and P7 L168-171:When the authors are able to just provide a figure or drawing to illustrate the ECC and the blood flow during the procedure, as this could benefit understanding for readers not too familiar with ECC-proceedings?

*🡪 We agree that a schematic illustration would substantially aid readers less familiar with the procedure. We have added a new figure (Figure X) schematically depicting the chemosaturation circuit: the hepatic arterial infusion catheter, the double-balloon catheter isolating the hepatic venous outflow, aspiration of hepatic venous blood through the catheter fenestrations into the extracorporeal circuit, passage through the activated charcoal filter, and return of the purified blood via the right internal jugular vein. The figure indicates that, during occlusion, venous return from the lower body reaches the right atrium exclusively via the extracorporeal circuit.*

Introduction/Methods: For instance, it is not completely clear enough whether during the balloon occlusion blood form the lower extremities, i.e. organs below liver/abdominal could still reach the right atrium, by an installed bypass bypassing the 2 ballons (connection temporary IVC-RA?). When not, then only the reduction of liver outflow+ the loss of preload from extremities and abdomen would be able to at least decrease cardiac output (C.O) by >20%.

*🡪 During balloon occlusion, the only venous return from the lower body reaching the right atrium is the blood routed through the extracorporeal circuit at the set flow rate of 500 mL/min. Venous return and cardiac preload from the entire infra-occlusion compartment (lower extremities, abdomen, splanchnic and renal beds) are therefore markedly reduced during the ECC phase. We agree that this preload reduction is expected to lower cardiac output substantially and contributes to the observed instability. We have clarified the flow path in the Methods and now present preload reduction and filter-mediated noradrenaline removal as two complementary, co-occurring mechanisms in the Discussion.
Currently we are conducting an in-vitro study to quantify the residual blood flow in the V. cava from the lower part of the body to the right atrium during balloon occlusion. Our goal is to quantify the residual leak in the system. So far, we have not detected any clinically relevant leakage. Results are expected to be published in spring 2027.*

Did you measure this by using apparatus to keep track on circulatory parameters like SVR/PAP, and or C.O.?

*🡪 We did not measure cardiac output, systemic vascular resistance, or pulmonary artery pressures. Hemodynamic monitoring comprised MAP, heart rate, central venous pressure, pulse pressure variation, and perfusion index. We have added the absence of cardiac output and SVR monitoring as a limitation. We note, however, that the filter effect is not reducible to preload loss: at the post-filter time point (T2.2), measured noradrenaline concentrations were lowest of the entire procedure (median 1017 pmol/L) despite the highest noradrenaline infusion rate (median 0.35 µg/kg/min). This dissociation between administered dose and circulating concentration cannot be explained by reduced preload, which would not lower the plasma concentration of infused noradrenaline, but is consistent with active trans-filter extraction.*

Were melphalan levels also measure pre- and post-ECC by HPLC also?

*🡪 Melphalan concentrations were not measured in this study. Our analyses focused on catecholamine kinetics and hemodynamic and metabolic parameters. We note that the melphalan filtration efficiency of the filter has been characterized in a porcine model (mean 99.0 ± 0.4%; Moeslein et al., Cardiovasc Intervent Radiol 2014).*

What are the possible differences betweenT1/2 of norepinephrine vs. epinephrine and dopamine, and could this have influenced the results?

*🡪 Noradrenaline (but not adrenaline or dopamine) was administered exogenously in relevant doses in anticipation of falling MAP and as a response to filtration triggered distributive shock. Therefore, noradrenaline concentrations are different in T1 and T2. As detailed in our response to Reviewer 2, the selectivity of extraction might partly be explained by these markedly different baseline concentrations (pre-filter noradrenaline was approximately 100-fold higher than adrenaline and dopamine) where a proportional reduction might not be reliably detected. We have expanded the Discussion accordingly.*

P6 here L144-148 all the exclusion criteria are listed. For instance pheochromocytoma was an exclusion. Of course anybody will understand this exogenous source of uncontrollable inotropic levels. Is it the case, that you only include patients with uveal melanoma (as stated in Table1)? Then this was maybe part of the inclusion criteria?

*🡪 In this cohort, all 15 procedures were performed in patients with uveal melanoma, as reflected in Table 1. This was not a formal inclusion criterion. It reflects the indication spectrum referred for chemosaturation at our center during the study period. We have clarified this in the Methods to avoid ambiguity.*

P 7L176. How was the blood flow of 500mL/min determined to be the right study-flow?

*🡪 The flow rate of 500 mL/min was not study-specific but corresponds to our standard institutional setting within the manufacturer-recommended range. The GLP validation of the identical GEN 2 filter used flow rates between 400 and 750 mL/min (mean 530 ± 34 mL/min; Moeslein et al. 2014), confirming that 500 mL/min lies within the validated operating range. We have added this clarification and reference to the Methods.*

P7L `184 You mean and eventually come to the conclusion that the adrenaline and dopamine measured were all exogenously released, due to the procedure and were not part of the medication/inotropic exogenous continuous support, see/conform L 178?

*🡪 That is correct. Only noradrenaline and vasopressin were administered as vasoactive agents. Neither adrenaline nor dopamine was given exogenously at any point. The adrenaline and dopamine concentrations measured therefore reflect endogenous levels only. We have made this explicit in the Methods and Results.*

P 17 Table 2 and P8/9 How did these levels of noradrenaline at 3 become 4-times levels of T1 and 20-times levels of T0? And secondly, how did the anesthesia team respond to possible hemodynamic support, when preload was restored?

*🡪 The marked rise in noradrenaline at T3 reflects the rebound that occurs once the activated charcoal filter is bypassed. During the filtration phase, continuously released endogenous and infused exogenous noradrenaline is adsorbed by the filter, keeping circulating concentrations low despite high infusion rates. Once filtration ends, this adsorption is removed while both endogenous release (driven by ongoing vasodilatory shock) and exogenous infusion (in lower doses) continue, leading to rapid accumulation. This rebound also provides indirect confirmation that active noradrenaline extraction occurred during filtration. We have clarified this in the Discussion. Shortly before termination of the extracorporeal circulation and restoration of caval venous return, vasopressor support was already lowered in anticipation of recovering hemodynamics. The transient post-filtration noradrenaline rebound described above therefore occurred despite, not because of, down-titration of exogenous noradrenaline, underscoring its mechanistic origin.*

P18 Table 4 and P9 How was PPV monitored/ Please describe and adapt , when possible? Was C.O. monitored?

*🡪 Pulse pressure variation was derived from the arterial pressure waveform by the patient monitor in mechanically ventilated patients. Cardiac output was not measured. We have specified the PPV source in the Methods and added the absence of cardiac output monitoring to the limitations.*

P9 As lactate levels rose to a maximum observed at T3, this may be a surrogate for tissue perfusion, was lactate clearance in anyway monitored? Or did you observe any differences in time for inotropic support after T3.

*🡪 No data were collected beyond T3. The study was deliberately focused on the intra-procedural mechanistic characterization of the filtration-associated shock, and no post-procedural follow-up of lactate clearance, acid-base normalization, or time to vasopressor independence was performed. We have stated this explicitly as a limitation. For the post-procedural course and outcome of chemosaturation patients at our center, we refer to our previous study (Rehn et al., Cancers 2024), in which peri-interventional lactate kinetics, coagulation recovery, and complications were analyzed in a larger cohort.*

In other words a surrogate for liver perfusion, that was seriously compromised by reduction of outflow, where inflow by the portal vein was still active and may be 70-80% of total liver flow. Therefore, you may not have seen changes in glucose levels and only in bicarbonate levels?
How fast did pH- and/or bicarbonate levels normalize after restoration of the liver outflow?

*🡪 We thank the Reviewer for this insightful hypothesis. We agree that preserved portal venous inflow during hepatic venous isolation likely maintains a substantial proportion of hepatic perfusion, which may explain why predominantly acid-base parameters (bicarbonate/base excess) rather than hepatic synthetic or metabolic functions were affected. We are, however, unable to test this directly: although blood glucose was measured clinically during the procedures, it was not systematically recorded for this study and therefore cannot be analyzed. However, in our clinical practice we have never seen clinically relevant liver failures after these procedures.*

What was the duration (min.) between T and T3?

*🡪 Procedure and inter-timepoint time intervals were not systematically recorded in the study dataset and therefore cannot be reported precisely. As a general reference, the median total procedure duration at our center is approximately 180 minutes (Rehn et al., Cancers 2024).*

P10 L286/287 Did you have contact or inform the manufacturer of the ECC-equipment used , as there may be important information available from animal- or preclinical studies of the properties of the filter, i.e. what is filtered and what is not and for which of these products this may be more flow-dependent?

*🡪 We thank the Reviewer for this valuable suggestion. We did contact the manufacturer (Delcath Systems) regarding preclinical in vitro data on the adsorption characteristics of the filter. The manufacturer confirmed that such data exist but was unable to share them, as they remain unpublished and proprietary. We have therefore based our mechanistic discussion on the available peer-reviewed preclinical evidence. The porcine study validating the filter system (Moeslein et al. 2014) explicitly reported that the hemofiltration cartridges adsorb non-specifically and demonstrated measurable reductions in multiple blood constituents (albumin, platelets, neutrophils, fibrinogen) beyond melphalan, attributed in part to filter adsorption rather than dilution alone. Notably, the same authors explicitly called for future studies to evaluate catecholamine extraction to better understand peri-procedural blood pressure management. We have incorporated this context into the Discussion.*

This question may also be seen in relation to your statement on P11 L326 “awareness of the selective nature of catecholamine removal may inform the development of procedure-specific hemodynamic algorithms, including proactive vasopressor combinations, careful volume management, and vigilant monitoring during transitions into and out of ECC” Please comment and adapt, when necessary.

*🡪 We have revised this passage to make clear that we do not propose a validated algorithm but rather argue that the mechanistic findings support a rationale for procedure-specific, vasopressor-oriented hemodynamic management. A specific strategy combining noradrenaline and vasopressin with balanced crystalloids has been described and evaluated in our previous cohort (Rehn et al., Cancers 2024).*

P12 L343-346 Time to normalization may have clinical effect on the outcome in the first days after he procedure, see above and take into regard my previous question?

*🡪 We agree that the time course of hemodynamic and metabolic normalization may influence early postoperative outcomes. As the present study did not include post-procedural follow-up, we cannot relate normalization kinetics to clinical outcomes within this dataset. We note that early postoperative outcomes, including lactate clearance and complication rates, were reported in our previous cohort (Rehn et al., Cancers 2024). Prospective studies linking intra-procedural mechanisms to outcomes remain warranted.*
