# Supplementary material for: Activated charcoal filtration profoundly alters systemic noradrenaline concentrations during hepatic chemosaturation: A prospective cohort study
Source: PLoS One. 2026 Aug 31;21(8):e0350771. doi: 10.1371/journal.pone.0350771 (PMC13528978; doi:10.1371/journal.pone.0350771)
Supplement: Supplementary file 6 [file pone.0350771.s007.docx]

Reviewer #3: Dear colleague,
here you receive my comment regarding the manuscript with the title “Activated charcoal filtration profoundly alters systemic noradrenaline concentrations during hepatic chemosaturation: a prospective cohort study”. With number PONE-D-26-16601R1.
The authors did their best to address all questions and adapted their manuscript where asked and necessary. Reading the manuscript again, I still come to a few remarks.

The extraction/filtration is not too medication specific, and heparin being used for anticoagulation during the procedure. How was the possible reduction in heparin by the absorbtion of the charcoal managed and monitored/measured?

*🡪 Thank you for this thoughtful comment. We now clarify the procedure in the manuscript. ACT was checked every 30 minutes during ECC to assure ACT was >400s. If ACT approached 400s or fell below 400s another bolus of 100 IU/kg heparin was administered and ACT was checked again.*

P7… a bolus of 400 IU/kg heparin was …ACT) > 450 s. … a second bolus of 100–200 IU/kg …,.. ACT was checked again. Ho w many patients needed this extra or 2nd bolus?

*🡪 Again, thank you for this comment. Unfortunately, we did not systematically record heparin doses in our dataset. However, ECC was closely monitored by the perfusionist and was not started if ACT was below 400s.*

P11 You write that “ … while lactate concentrations increased markedly, reaching a median of 26.4 mg/dl at T3 (all p < 0.001)….”’ Please explain what possibly induced the generation of the lactate to a median of 26.4 mg/dL (i.e. approx.. 2.93 mmol/L. Which may be considered clinically a mild increase? Was it the ischemia of the liver, the possible reduction in outflow of the lactate from the liver, or the influence of the oncolytic medication on the aerobe metabolism or other?

*🡪 We thank the reviewer for this valuable comment. We agree that our original discussion touched on this aspect only in general terms and did not sufficiently distinguish between the possible mechanisms the reviewer rightly raises. We have therefore substantially expanded the corresponding paragraph in the Discussion (P15). We have also followed the reviewer's implicit point regarding the magnitude of the change. The absolute lactate levels observed correspond to a mild increase, and we have adjusted the wording in both the Results and Discussion accordingly to avoid overstating the clinical severity. We hope the revised passage now adequately reflects the multifactorial nature of the observed hyperlactatemia.*


Typo’s: Page 3 Line 48. Please replace Capital for Noradrenaline, see e.g., L 47 and 50?
P5 L123 comparable thing here “…Hemodynamic instability…”change into “…hemodynamic…”P6”L150 “…noreadrenaline,… remove the ë”
Methods P7 There is no unity in writing the medication with a Capital letter or not, e.g., induction medication using Capital letters, heparin and melphalan there is not not and for Protamine (L188) , The Capital “P” is used again. Please reconsider and choose?
See P7 L176 and 177 for Heparin vs heparin

*🡪 We now addressed all mentioned typographical errors.*
