# Supplementary material for: Activated charcoal filtration profoundly alters systemic noradrenaline concentrations during hepatic chemosaturation: A prospective cohort study
Source: PLoS One. 2026 Aug 31;21(8):e0350771. doi: 10.1371/journal.pone.0350771 (PMC13528978; doi:10.1371/journal.pone.0350771)
Supplement: Peer review history

# Peer review: Decision Letter 0

**DOI:** 10.1371/journal.pone.0350771.r001

**Authors:** Jie Yang

23 Jun 2026

Dear Dr. Rehn,

Thank you for submitting your manuscript to PLOS ONE. After careful consideration, we feel that it has merit but does not fully meet PLOS ONE’s publication criteria as it currently stands. Therefore, we invite you to submit a revised version of the manuscript that addresses the points raised during the review process.

Please submit your revised manuscript by Aug 07 2026 11:59PM. If you will need more time than this to complete your revisions, please reply to this message or contact the journal office at plosone@plos.org. When you're ready to submit your revision, log on to https://www.editorialmanager.com/pone/ and select the 'Submissions Needing Revision' folder to locate your manuscript file.

- A letter that responds to each point raised by the academic editor and reviewer(s). You should upload this letter as a separate file labeled 'Response to Reviewers'.
- A marked-up copy of your manuscript that highlights changes made to the original version. You should upload this as a separate file labeled 'Revised Manuscript with Track Changes'.
- An unmarked version of your revised paper without tracked changes. You should upload this as a separate file labeled 'Manuscript'.

If you would like to make changes to your financial disclosure, please include your updated statement in your cover letter. Guidelines for resubmitting your figure files are available below the reviewer comments at the end of this letter.

If applicable, we recommend that you deposit your laboratory protocols in protocols.io to enhance the reproducibility of your results. Protocols.io assigns your protocol its own identifier (DOI) so that it can be cited independently in the future. For instructions see: https://journals.plos.org/plosone/s/submission-guidelines#loc-laboratory-protocols. Additionally, PLOS ONE offers an option for publishing peer-reviewed Lab Protocol articles, which describe protocols hosted on protocols.io. Read more information on sharing protocols at https://plos.org/protocols?utm_medium=editorial-email&utm_source=authorletters&utm_campaign=protocols.

As the corresponding author, your ORCID iD is verified in the submission system and will appear in the published article. PLOS supports the use of ORCID, and we encourage all coauthors to register for an ORCID iD and use it as well. Please encourage your coauthors to verify their ORCID iD within the submission system before final acceptance, as unverified ORCID iDs will not appear in the published article. *Only*  the individual author can complete the verification step; PLOS staff *cannot*  verify ORCID iDs on behalf of authors.

We look forward to receiving your revised manuscript.

Kind regards,

Jie Yang, M.D.

Guest Editor

PLOS One

Journal Requirements:

When submitting your revision, we need you to address these additional requirements.

1.Please ensure that your manuscript meets PLOS ONE's style requirements, including those for file naming. The PLOS ONE style templates can be found at

https://journals.plos.org/plosone/s/file?id=wjVg/PLOSOne_formatting_sample_main_body.pdf and

https://journals.plos.org/plosone/s/file?id=ba62/PLOSOne_formatting_sample_title_authors_affiliations.pdf

2. In the online submission form you indicate that your data is not available for proprietary reasons and have provided a contact point for accessing this data. Please note that your current contact point is a co-author on this manuscript. According to our Data Policy, the contact point must not be an author on the manuscript and must be an institutional contact, ideally not an individual. Please revise your data statement to a non-author institutional point of contact, such as a data access or ethics committee, and send this to us via return email. Please also include contact information for the third party organization, and please include the full citation of where the data can be found.

3. Please include captions for your Supporting Information files at the end of your manuscript, and update any in-text citations to match accordingly. Please see our Supporting Information guidelines for more information: http://journals.plos.org/plosone/s/supporting-information.

4. Thank you for providing your underlying data as Supporting Information.

We note that the data set contains text or data that is not in English. Please note that PLOS is an English-language publisher, so we require data sets to be provided in English as well. Please upload an English-language version of your data set.

This will also allow us to determine if your data follows PLOS standards per our Data Availability policy here: https://journals.plos.org/plosone/s/data-availability

5. Please upload a copy of your study protocol that was approved by your ethics committee/IRB as a Supporting Information file. By the study protocol, we mean the complete and detailed plan for the conduct and analysis of the trial approved by the ethics committee/IRB. Please send this in the original language. If this is in a language other than English, please also provide a translation. [https://journals.plos.org/plosone/s/submission-guidelines#loc-guidelines-for-specific-study-types

6. If the reviewer comments include a recommendation to cite specific previously published works, please review and evaluate these publications to determine whether they are relevant and should be cited. There is no requirement to cite these works unless the editor has indicated otherwise.

[Note: HTML markup is below. Please do not edit.]

Reviewers' comments:

Reviewer's Responses to Questions

**Comments to the Author**

1. Is the manuscript technically sound, and do the data support the conclusions?

Reviewer #1: Partly

Reviewer #2: Yes

Reviewer #3: Yes

**********

2. Has the statistical analysis been performed appropriately and rigorously?

Reviewer #1: Yes

Reviewer #2: Yes

Reviewer #3: Yes

**********

3. Have the authors made all data underlying the findings in their manuscript fully available?

The PLOS Data policy

Reviewer #1: Yes

Reviewer #2: Yes

Reviewer #3: Yes

**********

4. Is the manuscript presented in an intelligible fashion and written in standard English?

Reviewer #1: Yes

Reviewer #2: Yes

Reviewer #3: Yes

**********

Reviewer #1: This is a prospective, single-center observational study in which systemic catecholamine concentrations were measured in fifteen procedures undergoing hepatic chemo saturation. The objective appears to be determining if the activated charcoal filter used during chemo saturation absorb catecholamines leading to profound hemodynamic instability?

The primary outcome was the within-procedure change in catecholamine concentrations across the activated charcoal filter, quantified by paired pre-filter and post-filter plasma levels of noradrenaline, adrenaline, and dopamine during ECC (times T2.1 vs. T2.2).

There were five secondary outcomes.

1. Please determine if multiple comparison adjustments to the p-values for the multiple secondary outcomes are needed. All tables appear to be within any adjustment needed.

2. There was no motivation for sample size , this being an observational study. However, it would have been helpful to determine the adequacy of the sample size withing margin of error expectations as is not uncommon for this type of research and since inferential p-values are presented. They included 15 chemo saturation procedures in 12 individual patients in the analysis.

The statistical analysis is routine for the small sample consisting of continuous variables e reported as non parametric median and interquartile range (IQR). Categorical variables are reported as absolute and relative frequencies. Differences in catecholamine levels before and after filtration were analyzed using inferential non-parametric methods. For comparisons across multiple time points, the Friedman test was applied. For pairwise comparisons between pre- and post-filtration measurements, the Wilcoxon signed-rank test was used. To estimate the magnitude of the paired differences, the Hodges–Lehmann estimator with corresponding 95% confidence intervals (CI) was calculated. All the analyses were appropriate and well applied.

The paper was well written from a statistical perspective and the tables and figures were well formatted and interpretable.

The limitations section is incomplete.

3. Since the sample is so small a warning should be included with the limitations that this study is more observational with small sample size. Thus, the p-values should be interpreted with caution.

Reviewer #2: The manuscript addresses a clinically relevant question related to hemodynamic instability during hepatic chemosaturation. The topic is of interest, as it attempts to provide a mechanistic explanation for a well-recognized clinical phenomenon. The study is generally well organized and based on prospective data collection.

The manuscript does not provide a justification for the sample size. It appears that this study was conducted as an exploratory observational cohort without a formal sample size calculation. The authors should clarify whether a priori power analysis was performed. If not, this should be explicitly stated, and the exploratory nature of the study should be emphasized. Please provide a detailed explanation of the mechanism. The possible reasons why it only affects the concentration of norepinephrine need to be further elaborated.

Several minor language and formatting issues should be addressed. For example, an incorrect date is reported (“31.11.2025”), and there are inconsistencies in terminology (noradrenaline vs. norepinephrine). Additionally, minor formatting issues such as missing spaces between values and units (e.g., “16mmHg”) and incorrect confidence interval ordering should be corrected. These issues do not affect the scientific content but should be revised for clarity and accuracy.

Reviewer #3: Dear authors,

here you receive my comment regarding the manuscript with the title “Activated charcoal filtration profoundly alters systemic noradrenaline concentrations during hepatic chemosaturation: a prospective cohort study”. With number PONE-D-26-16601.

The authors present their results of clinical trial. The study was approved by the local medical ethical committee with the number provided and the study was registered with the German Clinical Trials Registry.

The article is well described and the question” Does the activated charcoal filter used during chemosaturation absorb catecholamines leading to profound hemodynamic instability? ”is studied and answered.

The study is presented straight forward and easy to understand. However, I have some remarks, that maybe after answering or leading to adaptions my improve further understanding.

PGE 5 Line 105-114 and P7 L168-171:When the authors are able to just provide a figure or drawing to illustrate the ECC and the blood flow during the procedure, as this could benefit understanding for readers not too familiar with ECC-proceedings?

Introduction/Methods: For instance, it is not completely clear enough whether during the balloon occlusion blood form the lower extremities, i.e. organs below liver/abdominal could still reach the right atrium, by an installed bypass bypassing the 2 ballons (connection temporary IVC-RA?). When not, then only he reduction of liver outflow+ the loss of preload from extremities and abdomen would be able to at least decrease cardiac output (C.O) by >20%. Did you measure this by using apparatus tot keep track on circulatory parameters like SVR/PAP, and or C.O.?

Were melphalan levels also measure pre- and post-ECC by HPLC also?

What are the possible differences betweenT1/2 of norepinephrine vs. epinephrine and dopamine, and could this have influenced the results?

P6 here L144-148 all the exclusion criteria are listed. For instance pheochromocytoma was an exclusion. Of course anybody will understand this exogenous source of uncontrollable inotropic levels. Is it the case, that you only include patients with uveal melanoma (as stated in Table1)? Then this was maybe part of the inclusion criteria?

P 7L176. How was the blood flow of 500mL/min determined to be the right study-flow?

P7L `184 You mean and eventually come to the conclusion that the adrenaline and dopamine measured were all exogenously released, due to the procedure and were not part of the medication/inotropic exogenous continuous support, see/conform L 178?

P 17 Table 2 and P8/9 How did these levels of noradrenaline at 3 become 4-times levels of T1 and 20-times levels of T0? And secondly, how did the anesthesia team respond to possible hemodynamic support, when preload was restored?

P18 Table 4 and P9 How was PPV monitored/ Please describe and adapt , when possible? Was C.O. monitored?

P9 As lactate levels rose to a maximum observed at T3, this may be a surrogate for tissue perfusion, was lactate clearance in anyway monitored? Or did you observe any differences in time for inotropic support after T3.

In other words a surrogate for liver perfusion, that was seriously compromised by reduction of outflow, where inflow by the portal vein was still active and may be 70-80% of total liver flow. Therefore, you may not have seen changes in glucose levels and only in bicarbonate levels?

How fast did pH- and/or bicarbonate levels normalize after restoration of the liver outflow?

What was the duration (min.) between T and T3?

P10 L286/287 Did you have contact or inform the manufacturer of the ECC-equipment used , as there may be important information available from animal- or preclinical studies of the properties of the filter, i.e. what is filtered and what is not and for which of these products this may be more flow-dependent?

This question may also be seen in relation to your statement on P11 L326 “awareness of the selective nature of catecholamine removal may inform the development of procedure-specific hemodynamic algorithms, including proactive vasopressor combinations, careful volume management, and vigilant monitoring during transitions into and out of ECC” Please comment and adapt, when necessary.

P12 L343-346 Time to normalization may have clinical effect on the outcome in the first days after he procedure, see above and take into regard my previous question?

**********

what does this mean?). If published, this will include your full peer review and any attached files.

If you choose “no”, your identity will remain anonymous but your review may still be made public.

**Do you want your identity to be public for this peer review?**  For information about this choice, including consent withdrawal, please see our Privacy Policy

Reviewer #1: No

Reviewer #2: No

Reviewer #3: **Yes: ** P.Bruins

**********

[NOTE: If reviewer comments were submitted as an attachment file, they will be attached to this email and accessible via the submission site. Please log into your account, locate the manuscript record, and check for the action link "View Attachments". If this link does not appear, there are no attachment files.]

To ensure your figures meet our technical requirements, please review our figure guidelines: https://journals.plos.org/plosone/s/figures

You may also use PLOS’s free figure tool, NAAS, to help you prepare publication quality figures: https://journals.plos.org/plosone/s/figures#loc-tools-for-figure-preparation.

NAAS will assess whether your figures meet our technical requirements by comparing each figure against our figure specifications.

# Author comment: Author response to Decision Letter 1

**DOI:** 10.1371/journal.pone.0350771.r002

5 Jul 2026

5. Review Comments to the Author

Reviewer #1: This is a prospective, single-center observational study in which systemic catecholamine concentrations were measured in fifteen procedures undergoing hepatic chemo saturation. The objective appears to be determining if the activated charcoal filter used during chemo saturation absorb catecholamines leading to profound hemodynamic instability?

The primary outcome was the within-procedure change in catecholamine concentrations across the activated charcoal filter, quantified by paired pre-filter and post-filter plasma levels of noradrenaline, adrenaline, and dopamine during ECC (times T2.1 vs. T2.2).

There were five secondary outcomes.

We thank Reviewer 1 for the careful and encouraging assessment of our manuscript and for confirming the appropriateness of the statistical approach. We have addressed all three points and provide additional analyses where requested.

1. Please determine if multiple comparison adjustments to the p-values for the multiple secondary outcomes are needed. All tables appear to be within any adjustment needed.

We thank the Reviewer for raising this important point. Our study was designed as a prospective, exploratory cohort study. The primary outcome was prespecified as the within-procedure trans-filter change in catecholamine concentrations (T2.1 vs. T2.2), whereas the secondary outcomes were hypothesis-generating. In line with established methodological recommendations for exploratory studies (Bender & Lange, J Clin Epidemiol 2001), a formal adjustment for multiplicity is therefore not mandatory, provided the exploratory nature is clearly stated. We have now made this explicit in the Statistical Methods and Limitations sections.

To nonetheless demonstrate the robustness of our findings, we additionally applied Bonferroni, Holm and Benjamini-Hochberg (false discovery rate) corrections to all primary and secondary tests. None of our conclusions changed: the trans-filter reduction of noradrenaline remained highly significant (Holm-adjusted p = 1.8 × 10⁻⁴), as did the noradrenaline time course (Holm-adjusted p = 4.0 × 10⁻⁸) and all eleven hemodynamic and metabolic secondary outcomes (all Holm-adjusted p < 0.008). The non-significant results for adrenaline and dopamine (both trans-filter and over time) were non-significant before and after adjustment.

2. There was no motivation for sample size , this being an observational study. However, it would have been helpful to determine the adequacy of the sample size withing margin of error expectations as is not uncommon for this type of research and since inferential p-values are presented. They included 15 chemo saturation procedures in 12 individual patients in the analysis.

We thank the Reviewer for this comment. As an exploratory observational study, no a priori sample size calculation was performed. Instead, we consecutively included all eligible chemosaturation procedures performed during the predefined study period. We have now stated this explicitly in the Methods.

Regarding the adequacy of the sample size: Across the 15 procedures, the trans-filter noradrenaline extraction rate was highly consistent (median 95.5%, distribution-free 95% CI 93.6–98.1%), and a reduction was observed in every single procedure (15/15), with a minimum extraction of 90.8%. As an additional precision estimate, the mean extraction rate was 95.6% (95% CI 94.3–97.0%), corresponding to a margin of error of ±1.3 percentage points. This indicates that, for the primary outcome, the sample of 15 procedures yields a precise and stable estimate. Even a sign test, which disregards effect magnitude entirely, yields p = 6.1 × 10⁻⁵ based on this concordance alone. This indicates that, for the primary outcome, the sample of 15 procedures provides a precise and stable estimate. By contrast, adrenaline (5 decreased / 5 increased / 5 unchanged) and dopamine (7/6/2) showed no directional effect, confirming that their non-significant results reflect a true absence of absorption rather than insufficient power. We have added a sentence summarizing the precision of the primary estimate to the Results and addressed the absence of an a priori power calculation in the Limitations.

The statistical analysis is routine for the small sample consisting of continuous variables e reported as non parametric median and interquartile range (IQR). Categorical variables are reported as absolute and relative frequencies. Differences in catecholamine levels before and after filtration were analyzed using inferential non-parametric methods. For comparisons across multiple time points, the Friedman test was applied. For pairwise comparisons between pre- and post-filtration measurements, the Wilcoxon signed-rank test was used. To estimate the magnitude of the paired differences, the Hodges–Lehmann estimator with corresponding 95% confidence intervals (CI) was calculated. All the analyses were appropriate and well applied.

The paper was well written from a statistical perspective and the tables and figures were well formatted and interpretable.

The limitations section is incomplete.

3. Since the sample is so small a warning should be included with the limitations that this study is more observational with small sample size. Thus, the p-values should be interpreted with caution.

We agree and have expanded the Limitations section to emphasize the small, single-center sample, the exploratory design, the absence of an a-priori sample size calculation, and the resulting need to interpret the p-values of the secondary outcomes as hypothesis-generating.

Reviewer #2: The manuscript addresses a clinically relevant question related to hemodynamic instability during hepatic chemosaturation. The topic is of interest, as it attempts to provide a mechanistic explanation for a well-recognized clinical phenomenon. The study is generally well organized and based on prospective data collection.

We thank Reviewer 2 for the positive and constructive evaluation of our manuscript and for recognizing the clinical relevance of the research question and the value of our prospective data collection. We appreciate the helpful suggestions regarding the sample size justification, the mechanistic interpretation, and several formal points. We have addressed all comments in detail below and revised the manuscript accordingly.

The manuscript does not provide a justification for the sample size. It appears that this study was conducted as an exploratory observational cohort without a formal sample size calculation. The authors should clarify whether a priori power analysis was performed. If not, this should be explicitly stated, and the exploratory nature of the study should be emphasized.

We thank the Reviewer for this comment. The Reviewer is correct: this was a prospective, exploratory observational cohort study, and no a priori power analysis or formal sample size calculation was performed. Instead, we consecutively included all eligible chemosaturation procedures performed at our center during the predefined study period. We have now stated this explicitly in the Methods and have emphasized the exploratory nature of the study in both the Methods and the Limitations sections.

To address the adequacy of the available sample, we additionally evaluated the precision of the primary effect estimate. Across the 15 procedures, the trans-filter noradrenaline extraction rate was highly consistent (median 95.5%, distribution-free 95% CI 93.6–98.1%), and a reduction was observed in every single procedure (15/15), with a minimum extraction of 90.8%. As an additional precision estimate, the mean extraction rate was 95.6% (95% CI 94.3–97.0%), corresponding to a margin of error of ±1.3 percentage points. This indicates that, for the primary outcome, the sample of 15 procedures yields a precise and stable estimate. By contrast, adrenaline and dopamine showed no directional trans-filter change, confirming that their non-significant results reflect a true absence of adsorption rather than insufficient statistical power. We have added a sentence summarizing the precision of the primary estimate to the Results, and we now explicitly address the absence of an a priori power calculation, the small single-center sample, and the resulting exploratory interpretation of the p-values in the Limitations section. We note that this point was also raised by Reviewer 1. The corresponding manuscript changes address both comments.

Please provide a detailed explanation of the mechanism. The possible reasons why it only affects the concentration of norepinephrine need to be further elaborated.

We thank the Reviewer for this important comment. We have substantially expanded the corresponding section of the Discussion and now offer two complementary explanations.

First, the apparent selectivity is to a relevant degree a consequence of the markedly different baseline concentrations of the three catecholamines. Pre-filter noradrenaline concentrations were approximately 100-fold higher (median 27,404 pmol/L) than those of adrenaline (median 274 pmol/L) and dopamine (median 448 pmol/L). This reflects the clinical setting, in which noradrenaline is both endogenously released under procedural stress and exogenously administered in high doses, whereas adrenaline and dopamine are neither supplemented nor substantially elevated. Throughout the procedure, adrenaline and dopamine concentrations remained in the low physiological range, where a proportional reduction across the filter cannot be reliably detected. Consequently, the pronounced trans-filter gradient observed for noradrenaline, and its absence for adrenaline and dopamine, is at least partly attributable to these differences in circulating concentration rather than to an exclusively substance-specific affinity of the activated charcoal. We now state this explicitly. Second, beyond this concentration effect, a genuinely substance-specific component cannot be excluded. Activated charcoal adsorbs solutes primarily through hydrophobic and π-π interactions, and small differences in molecular structure, polarity, and plasma protein binding among the three catecholamines may modulate their respective adsorption affinity. However, in the absence of dedicated in-vitro adsorption experiments, this physicochemical mechanism remains hypothetical, and we have framed it accordingly.

We believe that presenting both the concentration-dependent and the potential physicochemical contributions provides a more accurate and balanced mechanistic interpretation than attributing the effect to filter selectivity alone.

Several minor language and formatting issues should be addressed. For example, an incorrect date is reported (“31.11.2025”), and there are inconsistencies in terminology (noradrenaline vs. norepinephrine). Additionally, minor formatting issues such as missing spaces between values and units (e.g., “16mmHg”) and incorrect confidence interval ordering should be corrected. These issues do not affect the scientific content but should be revised for clarity and accuracy.

Thank you for this valuable comment. We now eliminated all inconsistencies and language errors.

Reviewer #3: Dear authors,

here you receive my comment regarding the manuscript with the title “Activated charcoal filtration profoundly alters systemic noradrenaline concentrations during hepatic chemosaturation: a prospective cohort study”. With number PONE-D-26-16601.

The authors present their results of clinical trial. The study was approved by the local medical ethical committee with the number provided and the study was registered with the German Clinical Trials Registry.

The article is well described and the question” Does the activated charcoal filter used during chemosaturation absorb catecholamines leading to profound hemodynamic instability? ”is studied and answered.

The study is presented straight forward and easy to understand. However, I have some remarks, that maybe after answering or leading to adaptions my improve further understanding.

We thank Reviewer 3 for the thorough and constructive evaluation of our manuscript and for the many detailed questions, which have helped us clarify the procedure and strengthen the mechanistic interpretation. We have addressed each point below and revised the manuscript accordingly.

PGE 5 Line 105-114 and P7 L168-171:When the authors are able to just provide a figure or drawing to illustrate the ECC and the blood flow during the procedure, as this could benefit understanding for readers not too familiar with ECC-proceedings?

We agree that a schematic illustration would substantially aid readers less familiar with the procedure. We have added a new figure (Figure X) schematically depicting the chemosaturation circuit: the hepatic arterial infusion catheter, the double-balloon catheter isolating the hepatic venous outflow, aspiration of hepatic venous blood through the catheter fenestrations into the extracorporeal circuit, passage through the activated charcoal filter, and return of the purified blood via the right internal jugular vein. The figure indicates that, during occlusion, venous return from the lower body reaches the right atrium exclusively via the extracorporeal circuit.

Introduction/Methods: For instance, it is not completely clear enough whether during the balloon occlusion blood form the lower extremities, i.e. organs below liver/abdominal could still reach the right atrium, by an installed bypass bypassing the 2 ballons (connection temporary IVC-RA?). When not, then only the reduction of liver outflow+ the loss of preload from extremities and abdomen would be able to at least decrease cardiac output (C.O) by >20%.

During balloon occlusion, the only venous return from the lower body reaching the right atrium is the blood routed through the extracorporeal circuit at the set flow rate of 500 mL/min. Venous return and cardiac preload from the entire infra-occlusion compartment (lower extremities, abdomen, splanchnic and renal beds) are therefore markedly reduced during the ECC phase. We agree that this preload reduction is expected to lower cardiac output substantially and contributes to the observed instability. We have clarified the flow path in the Methods and now present preload reduction and filter-mediated noradrenaline removal as two complementary, co-occurring mechanisms in the Discussion.

Currently we are conducting an in-vitro study to quantify the residual blood flow in the V. cava from the lower part of the body to the right atrium during balloon occlusion. Our goal is to quantify the residual leak in the system. So far, we have not detected any clinically relevant leakage. Results are expected to be published in spring 2027.

Did you measure this by using apparatus to keep track on circulatory parameters like SVR/PAP, and or C.O.?

We did not measure cardiac output, systemic vascular resistance, or pulmonary artery pressures. Hemodynamic monitoring comprised MAP, heart rate, central venous pressure, pulse pressure variation, and perfusion index. We have added the absence of cardiac output and SVR monitoring as a limitation. We note, however, that the filter effect is not reducible to preload loss: at the post-filter time point (T2.2), measured noradrenaline concentrations were lowest of the entire procedure (median 1017 pmol/L) despite the highest noradrenaline infusion rate (median 0.35 µg/kg/min). This dissociation between administered dose and circulating concentration cannot be explained by reduced preload, which would not lower the plasma concentration of infused noradrenaline, but is consistent with active trans-filter extraction.

Were melphalan levels also measure pre- and post-ECC by HPLC also?

Melphalan concentrations were not measured in this study. Our analyses focused on catecholamine kinetics and hemodynamic and metabolic parameters. We note that the melphalan filtration efficiency of the filter has been characterized in a porcine model (mean 99.0 ± 0.4%; Moeslein et al., Cardiovasc Intervent Radiol 2014).

What are the possible differences betweenT1/2 of norepinephrine vs. epinephrine and dopamine, and could this have influenced the results?

Noradrenaline (but not adrenaline or dopamine) was administered exoge

Attachment Submitted filename: Comments.docx

# Peer review: Decision Letter 1

**DOI:** 10.1371/journal.pone.0350771.r003

**Authors:** Jie Yang

15 Jul 2026

Dear Dr. Rehn,

Thank you for submitting your manuscript to PLOS ONE. After careful consideration, we feel that it has merit but does not fully meet PLOS ONE’s publication criteria as it currently stands. Therefore, we invite you to submit a revised version of the manuscript that addresses the points raised during the review process.

Please submit your revised manuscript by Aug 29 2026 11:59PM. If you will need more time than this to complete your revisions, please reply to this message or contact the journal office at plosone@plos.org. When you're ready to submit your revision, log on to https://www.editorialmanager.com/pone/ and select the 'Submissions Needing Revision' folder to locate your manuscript file.

- A letter that responds to each point raised by the academic editor and reviewer(s). You should upload this letter as a separate file labeled 'Response to Reviewers'.
- A marked-up copy of your manuscript that highlights changes made to the original version. You should upload this as a separate file labeled 'Revised Manuscript with Track Changes'.
- An unmarked version of your revised paper without tracked changes. You should upload this as a separate file labeled 'Manuscript'.

If you would like to make changes to your financial disclosure, please include your updated statement in your cover letter. Guidelines for resubmitting your figure files are available below the reviewer comments at the end of this letter.

If applicable, we recommend that you deposit your laboratory protocols in protocols.io to enhance the reproducibility of your results. Protocols.io assigns your protocol its own identifier (DOI) so that it can be cited independently in the future. For instructions see: https://journals.plos.org/plosone/s/submission-guidelines#loc-laboratory-protocols. Additionally, PLOS ONE offers an option for publishing peer-reviewed Lab Protocol articles, which describe protocols hosted on protocols.io. Read more information on sharing protocols at https://plos.org/protocols?utm_medium=editorial-email&utm_source=authorletters&utm_campaign=protocols.

As the corresponding author, your ORCID iD is verified in the submission system and will appear in the published article. PLOS supports the use of ORCID, and we encourage all coauthors to register for an ORCID iD and use it as well. Please encourage your coauthors to verify their ORCID iD within the submission system before final acceptance, as unverified ORCID iDs will not appear in the published article. *Only*  the individual author can complete the verification step; PLOS staff *cannot*  verify ORCID iDs on behalf of authors.

We look forward to receiving your revised manuscript.

Kind regards,

Jie Yang, M.D.

Guest Editor

PLOS One

Journal Requirements:

If the reviewer comments include a recommendation to cite specific previously published works, please review and evaluate these publications to determine whether they are relevant and should be cited. There is no requirement to cite these works unless the editor has indicated otherwise.

Please review your reference list to ensure that it is complete and correct. If you have cited papers that have been retracted, please include the rationale for doing so in the manuscript text, or remove these references and replace them with relevant current references. Any changes to the reference list should be mentioned in the rebuttal letter that accompanies your revised manuscript. If you need to cite a retracted article, indicate the article’s retracted status in the References list and also include a citation and full reference for the retraction notice.

[Note: HTML markup is below. Please do not edit.]

Reviewers' comments:

Reviewer's Responses to Questions

**Comments to the Author**

Reviewer #1: All comments have been addressed

Reviewer #2: All comments have been addressed

Reviewer #3: All comments have been addressed

**********

2. Is the manuscript technically sound, and do the data support the conclusions?

Reviewer #1: (No Response)

Reviewer #2: Yes

Reviewer #3: Yes

**********

3. Has the statistical analysis been performed appropriately and rigorously?

Reviewer #1: (No Response)

Reviewer #2: Yes

Reviewer #3: Yes

**********

4. Have the authors made all data underlying the findings in their manuscript fully available?

The PLOS Data policy

Reviewer #1: (No Response)

Reviewer #2: Yes

Reviewer #3: Yes

**********

5. Is the manuscript presented in an intelligible fashion and written in standard English?

Reviewer #1: (No Response)

Reviewer #2: Yes

Reviewer #3: Yes

**********

Reviewer #1: (No Response)

Reviewer #2: I appreciate the authors' efforts in revising the manuscript. Based on the revised version and the detailed point-by-point responses, I have no further comments. In my opinion, the manuscript is now suitable for publication in its current form.

Reviewer #3: Dear colleague,

here you receive my comment regarding the manuscript with the title “Activated charcoal filtration profoundly alters systemic noradrenaline concentrations during hepatic chemosaturation: a prospective cohort study”. With number PONE-D-26-16601R1.

The authors did their best to address all questions and adapted their manuscript where asked and necessary. Reading the manuscript again, I still come to a few remarks.

The extraction/filtration is not too medication specific, and heparin being used for anticoagulation during the procedure. How was the possible reduction in heparin by the absorbtion of the charcoal managed and monitored/measured?

P7… a bolus of 400 IU/kg heparin was …ACT) > 450 s. … a second bolus of 100–200 IU/kg …,.. ACT was checked again. Ho w many patients needed this extra or 2nd bolus?

P11 You write that “ … while lactate concentrations increased markedly, reaching a median of 26.4 mg/dl at T3 (all p < 0.001)….”’ Please explain what possibly induced the generation of the lactate to a median of 26.4 mg/dL (i.e. approx.. 2.93 mmol/L. Which may be considered clinically a mild increase? Was it the ischemia of the liver, the possible reduction in outflow of the lactate from the liver, or the influence of the oncolytic medication on the aerobe metabolism or other?

Typo’s: Page 3 Line 48. Please replace Capital for Noradrenaline, see e.g., L 47 and 50?

P5 L123 comparable thing here “…Hemodynamic instability…”change into “…hemodynamic…”P6”L150 “…noreadrenaline,… remove the ë”

Methods P7 There is no unity in writing the medication with a Capital letter or not, e.g., induction medication using Capital letters, heparin and melphalan there is not not and for Protamine (L188) , The Capital “P” is used again. Please reconsider and choose?

See P7 L176 and 177 for Heparin vs heparin

**********

what does this mean?). If published, this will include your full peer review and any attached files.

If you choose “no”, your identity will remain anonymous but your review may still be made public.

**Do you want your identity to be public for this peer review?**  For information about this choice, including consent withdrawal, please see our Privacy Policy

Reviewer #1: No

Reviewer #2: No

Reviewer #3: **Yes: ** P.Bruins

**********

[NOTE: If reviewer comments were submitted as an attachment file, they will be attached to this email and accessible via the submission site. Please log into your account, locate the manuscript record, and check for the action link "View Attachments". If this link does not appear, there are no attachment files.]

To ensure your figures meet our technical requirements, please review our figure guidelines: https://journals.plos.org/plosone/s/figures

You may also use PLOS’s free figure tool, NAAS, to help you prepare publication quality figures: https://journals.plos.org/plosone/s/figures#loc-tools-for-figure-preparation..

NAAS will assess whether your figures meet our technical requirements by comparing each figure against our figure specifications.

# Author comment: Author response to Decision Letter 2

**DOI:** 10.1371/journal.pone.0350771.r004

24 Jul 2026

Reviewer #3: Dear colleague,

here you receive my comment regarding the manuscript with the title “Activated charcoal filtration profoundly alters systemic noradrenaline concentrations during hepatic chemosaturation: a prospective cohort study”. With number PONE-D-26-16601R1.

The authors did their best to address all questions and adapted their manuscript where asked and necessary. Reading the manuscript again, I still come to a few remarks.

The extraction/filtration is not too medication specific, and heparin being used for anticoagulation during the procedure. How was the possible reduction in heparin by the absorbtion of the charcoal managed and monitored/measured?

-->Thank you for this thoughtful comment. We now clarify the procedure in the manuscript. ACT was checked every 30 minutes during ECC to assure ACT was >400s. If ACT approached 400s or fell below 400s another bolus of 100 IU/kg heparin was administered and ACT was checked again.

P7… a bolus of 400 IU/kg heparin was …ACT) > 450 s. … a second bolus of 100–200 IU/kg …,.. ACT was checked again. Ho w many patients needed this extra or 2nd bolus?

--> Again, thank you for this comment. Unfortunately, we did not systematically record heparin doses in our dataset. However, ECC was closely monitored by the perfusionist and was not started if ACT was below 400s.

P11 You write that “ … while lactate concentrations increased markedly, reaching a median of 26.4 mg/dl at T3 (all p < 0.001)….”’ Please explain what possibly induced the generation of the lactate to a median of 26.4 mg/dL (i.e. approx.. 2.93 mmol/L. Which may be considered clinically a mild increase? Was it the ischemia of the liver, the possible reduction in outflow of the lactate from the liver, or the influence of the oncolytic medication on the aerobe metabolism or other?

--> We thank the reviewer for this valuable comment. We agree that our original discussion touched on this aspect only in general terms and did not sufficiently distinguish between the possible mechanisms the reviewer rightly raises. We have therefore substantially expanded the corresponding paragraph in the Discussion (P15). We have also followed the reviewer's implicit point regarding the magnitude of the change. The absolute lactate levels observed correspond to a mild increase, and we have adjusted the wording in both the Results and Discussion accordingly to avoid overstating the clinical severity. We hope the revised passage now adequately reflects the multifactorial nature of the observed hyperlactatemia.

Typo’s: Page 3 Line 48. Please replace Capital for Noradrenaline, see e.g., L 47 and 50?

P5 L123 comparable thing here “…Hemodynamic instability…”change into “…hemodynamic…”P6”L150 “…noreadrenaline,… remove the ë”

Methods P7 There is no unity in writing the medication with a Capital letter or not, e.g., induction medication using Capital letters, heparin and melphalan there is not not and for Protamine (L188) , The Capital “P” is used again. Please reconsider and choose?

See P7 L176 and 177 for Heparin vs heparin

--> We now addressed all mentioned typographical errors.

Attachment Submitted filename: Comments2.docx

# Peer review: Decision Letter 2

**DOI:** 10.1371/journal.pone.0350771.r005

**Authors:** Jie Yang

12 Aug 2026

Activated charcoal filtration profoundly alters systemic noradrenaline concentrations during hepatic chemosaturation: a prospective cohort study

PONE-D-26-16601R2

Dear Dr. Rehn,

We’re pleased to inform you that your manuscript has been judged scientifically suitable for publication and will be formally accepted for publication once it meets all outstanding technical requirements.

Within one week, you’ll receive an e-mail detailing the required amendments. When these have been addressed, you’ll receive a formal acceptance letter and your manuscript will be scheduled for publication.

An invoice will be generated when your article is formally accepted. Please note, if your institution has a publishing partnership with PLOS and your article meets the relevant criteria, all or part of your publication costs will be covered. Please make sure your user information is up-to-date by logging into Editorial Manager at Editorial Manager® and clicking the ‘Update My Information' link at the top of the page. For questions related to billing, please contact billing support.

If your institution or institutions have a press office, please notify them about your upcoming paper to help maximize its impact. If they’ll be preparing press materials, please inform our press team as soon as possible -- no later than 48 hours after receiving the formal acceptance. Your manuscript will remain under strict press embargo until 2 pm Eastern Time on the date of publication. For more information, please contact onepress@plos.org.

Kind regards,

Jie Yang, M.D.

Guest Editor

PLOS One

Additional Editor Comments (optional):

Reviewers' comments:

Reviewer's Responses to Questions

**Comments to the Author**

Reviewer #1: All comments have been addressed

**********

2. Is the manuscript technically sound, and do the data support the conclusions?

Reviewer #1: (No Response)

**********

3. Has the statistical analysis been performed appropriately and rigorously?

Reviewer #1: (No Response)

**********

4. Have the authors made all data underlying the findings in their manuscript fully available?

The PLOS Data policy

Reviewer #1: (No Response)

**********

5. Is the manuscript presented in an intelligible fashion and written in standard English?

Reviewer #1: (No Response)

**********

Reviewer #1: (No Response)

**********

what does this mean?). If published, this will include your full peer review and any attached files.

If you choose “no”, your identity will remain anonymous but your review may still be made public.

**Do you want your identity to be public for this peer review?**  For information about this choice, including consent withdrawal, please see our Privacy Policy

Reviewer #1: No

**********

# Editor report: Acceptance letter

**DOI:** 10.1371/journal.pone.0350771.r006

**Authors:** Jie Yang

PONE-D-26-16601R2

PLOS One

Dear Dr. Rehn,

I'm pleased to inform you that your manuscript has been deemed suitable for publication in PLOS One. Congratulations! Your manuscript is now being handed over to our production team.

At this stage, our production department will prepare your paper for publication. This includes ensuring the following:

* All references, tables, and figures are properly cited

* All relevant supporting information is included in the manuscript submission,

* There are no issues that prevent the paper from being properly typeset

You will receive further instructions from the production team, including instructions on how to review your proof when it is ready. Please keep in mind that we are working through a large volume of accepted articles, so please give us a few days to review your paper and let you know the next and final steps.

Lastly, if your institution or institutions have a press office, please let them know about your upcoming paper now to help maximize its impact. If they'll be preparing press materials, please inform our press team within the next 48 hours. Your manuscript will remain under strict press embargo until 2 pm Eastern Time on the date of publication. For more information, please contact onepress@plos.org.

You will receive an invoice from PLOS for your publication fee after your manuscript has reached the completed accept phase. If you receive an email requesting payment before acceptance or for any other service, this may be a phishing scheme. Learn how to identify phishing emails and protect your accounts at https://explore.plos.org/phishing.

If we can help with anything else, please email us at customercare@plos.org.

Thank you for submitting your work to PLOS One and supporting open access.

Kind regards,

PLOS ONE Editorial Office Staff

on behalf of

Dr. Jie Yang

Guest Editor

PLOS One
